# The human LRRK2-R1441G mutation drives age-dependent oxidative stress and mitochondrial dysfunction in dopaminergic neurons

**DOI:** 10.1186/s13024-026-00969-7

**Published:** 2026-07-07

**Authors:** Yuanxin Chen, Lianteng Zhi, Shiquan Cui, Huaixin Wang, Chenbo Zeng, Hui Zhang

**Affiliations:** 1 Department of Physiology & Pharmacology, Isakson Center for Neurological Disease Research, College of Veterinary Medicine, The University of Georgia, 501 D.W. Brooks Drive, Athens, GA 30602 USA; 2 Department of Neuroscience, Thomas Jefferson University, Philadelphia, 19107 USA

**Keywords:** Parkinson’s disease, LRRK2, Mitochondrial dysfunction, Oxidative stress, Mitochondrial membrane potential (MMP), Uncoupling protein (UCP)

## Abstract

**Background:**

Mitochondrial dysfunction and oxidative stress are central to the pathogenesis of Parkinson’s disease (PD), particularly affecting substantia nigra pars compacta (SNc) dopamine (DA) neurons. Here, we investigate how the R1441G mutation in leucine-rich repeat kinase 2 (LRRK2), a key genetic contributor to familial and sporadic PD, impacts mitochondrial function in midbrain DA neurons.

**Methods:**

We employed a BAC transgenic mouse model overexpressing human LRRK2–R1441G (BAC-hR1441G) and crossed it with TH-mito-roGFP mice to enable mitochondria-targeted redox imaging specifically in DA neurons. Acute midbrain slices from 3-, 6-, and 12-month-old mice were imaged using two-photon microscopy to assess mitochondrial oxidative stress. In parallel, mitochondrial respiratory function, membrane potential flickering events, and expression of uncoupling proteins (UCP4/UCP5) were analyzed. Spatial transcriptomic profiling was performed using the GeoMx® Digital Spatial Profiler to uncover associated molecular alterations.

**Results:**

We observed a progressive increase in mitochondrial oxidative stress in SNc DA neurons of BAC-hR1441G mice at 3, 6, and 12 months of age. This was accompanied by reduced respiratory complex activity, attenuated mitochondrial membrane potential flickering, and diminished expression of UCP4 and UCP5. Spatial transcriptomic analysis revealed dysregulation of genes linked to mitochondrial uncoupling, calcium signaling, and redox regulation in BAC-hR1441G SNc DA neurons.

**Conclusions:**

These findings reveal an age-dependent progression of mitochondrial dysfunction in BAC-hR1441G SNc DA neurons. Dysregulation of calcium channels and uncoupling proteins emerges as a key mechanism contributing to bioenergetic failure, suggesting potential therapeutic targets to mitigate PD progression.

**Supplementary Information:**

The online version contains supplementary material available at https://doi.org/10.1186/s13024-026-00969-7.

## Background

Parkinson’s disease (PD) is a progressive neurodegenerative disorder characterized by the selective loss of dopaminergic (DA) neurons in the substantia nigra pars compacta (SNc) and the accumulation of α-synuclein pathology [1–3]. While both genetic and environmental factors contribute to PD, aging remains the greatest risk factor [4]. However, the mechanisms by which age-related cellular dysfunctions drive PD pathogenesis remain poorly understood. Among the most implicated factors, mitochondrial dysfunction and oxidative stress have emerged as critical contributors to dopaminergic neurodegeneration [5].

Mutations in leucine-rich repeat kinase 2 (LRRK2) are the most common genetic cause of both familial and sporadic PD [6–9]. Among these, the R1441G mutation disrupts the ROC GTPase domain, leading to enhanced LRRK2 kinase activity and downstream cellular dysfunction [10]. Previous studies have linked LRRK2 mutations, including R1441G, to multiple mitochondrial abnormalities, such as impaired mitochondrial respiration, altered mitochondrial morphology and dynamics, increased oxidative stress, disrupted calcium homeostasis, and defects in mitochondrial quality control [10–18]. These findings support a critical role for mitochondrial dysfunction in LRRK2-associated PD. However, most studies have relied on cultured cells, induced pluripotent stem cell-derived neurons, or biochemical analyses of bulk tissue. Consequently, the temporal progression of mitochondrial dysfunction within identified DA neurons during aging remains poorly understood. Furthermore, it remains unclear whether mitochondrial dysfunction occurs selectively within vulnerable nigrostriatal DA neurons or represents a more generalized phenomenon across neuronal populations.

Mitochondria are essential for ATP production, calcium buffering, and reactive oxygen species (ROS) regulation [19, 20]. This is particularly important in SNc DA neurons, which experience substantial bioenergetic stress due to their autonomous pace-making activity, extensive axonal arborization, and sustained calcium influx through L-type calcium channels. Previous work has suggested that mitochondrial oxidant stress and calcium dysregulation contribute to the selective vulnerability of these neurons in PD [21]. Notably, mitochondrial oxidative stress can be exacerbated by dysregulated calcium homeostasis, as excessive cytosolic and mitochondrial calcium levels further impair mitochondrial respiration and trigger cell death pathways [22, 23]. However, direct evidence linking the age-dependent development of mitochondrial oxidative stress to neuronal vulnerability in LRRK2-associated PD remains limited.

Here, we investigated mitochondrial dysfunction in a BAC transgenic mouse model expressing human LRRK2–R1441G, a model previously shown to develop age-dependent motor, neurochemical, and pathological abnormalities relevant to PD [24]. By crossing BAC-hR1441G mice with TH-mito-roGFP reporter mice and applying two-photon ratiometric imaging, we directly visualized age-dependent mitochondrial dysfunction in identified DA neurons and their striatal projections. We observed early mitochondrial oxidative stress in striatal DA terminals, accompanied by elevated mitochondrial calcium levels and increased mitochondrial calcium uniporter (MCU) expression. Pharmacological blockade of L-type calcium channels significantly reduced oxidative stress, supporting a role for calcium dysregulation in mitochondrial impairment. In addition, BAC-hR1441G DA neurons exhibited impaired mitochondrial membrane potential (MMP) flickering and reduced expression of UCP4 and UCP5, which may further compromise mitochondrial homeostasis and antioxidant capacity [5, 25]. Spatial transcriptomic analyses of the SN provided independent evidence for dysregulation of redox-related pathways in BAC-hR1441G DA neurons. Collectively, these findings extend previous studies [5, 25] linking LRRK2 mutations to mitochondrial dysfunction and provide an integrated characterization of age-dependent mitochondrial oxidative stress, calcium dysregulation, and mitochondrial homeostatic defects in vulnerable dopaminergic neurons, highlighting MCU, UCPs, and L-type calcium channels as potential therapeutic targets in Parkinson’s disease.

## Materials and methods

### Animals

BAC-hLRRK2-R1441G transgenic mice (Stock No. 009604, The Jackson Laboratory) and BAC-hLRRK2-WT transgenic mice (Stock No. 009610, The Jackson Laboratory) were obtained from Dr. Chenjian Li’s laboratory at Weill Medical College of Cornell University and maintained on FVB/N background (Taconic Biosciences). Transgenic mice expressing a roGFP construct under the tyrosine hydroxylase (TH) promoter, with a mitochondria-targeting matrix sequence, were acquired from Dr. James Surmeier’s laboratory at Northwestern University. These mice were backcrossed onto the FVB/N background for eight generations before being crossed with BAC-hR1441G mice, generating roGFP-expressing hR1441G and non-transgenic (nTg) lines. All mice were maintained on a congenic FVB/N background.

Both male and female mice were used and distributed across experimental groups at the indicated ages. Animals were housed under specific pathogen-free conditions in a temperature- and humidity-controlled facility on a 12-h light/dark cycle (07:00–19:00 light) with ad libitum access to food and water. Mice were randomly assigned to experimental groups whenever appropriate. Investigators were blinded to genotype during data acquisition and analysis whenever feasible. 

### Ethics declaration

All animal experiments were conducted in accordance with the National Institutes of Health guidelines. Experimental procedures were approved by the Institutional Animal Care and Use Committees (IACUC) of Thomas Jefferson University (Protocol *01452*) and the University of Georgia (Protocol $\# {\rm{ A}}2023{\rm{ }}05 - 031 - {\rm{Y}}1 - {\rm{A}}1$). All efforts were made to minimize animal suffering and to reduce the number of animals used.

### Brain slice preparation

Mice were anesthetized using a ketamine/xylazine mixture, followed by transcardial perfusion with ice-cold, oxygenated cutting solution containing (in mM): 125 NaCl, 2.5 KCl, 26 NaHCO_3_, 3.7 MgSO_4_, 0.3 KH_2_PO_4_, and 10 glucose (pH 7.4). Following perfusion, the mice were decapitated, and the brains were immediately removed and sectioned in ice-cold, oxygenated cutting solution using a vibratome (VT1000S, Leica Microsystems, Germany). Brain slices, 250 µm in thickness, were recovered at 34–35 °C in oxygenated artificial cerebrospinal fluid (aCSF; in mM: 125 NaCl, 2.5 KCl, 26 NaHCO_3_, 2.4 CaCl_2_, 1.3 MgSO_4_, 0.3 KH_2_PO_4_, 10 glucose, 2 HEPES, pH 7.4) for 30 min and then maintained at room temperature.

For oxygen consumption rate (OCR) measurements, 160 µm-thick slices of the striatum were prepared and transferred to respiration buffer composed of preoxygenated aCSF supplemented with 25 mM glucose, 2 mM L-glutamine, and 4 mg/mL bovine serum albumin (BSA), freshly added before use (pH 7.4, 37 °C). Tissue punches were immediately taken from the slices for subsequent respiration studies.

### TH Immunogold labeling and electron microscope

LRRK2 WT and BAC-hR1441G mice were perfused transcardially with 3.75% acrolein and 2% paraformaldehyde in 0.1 M sodium phosphate buffer (PB, pH 7.4). Brains were removed and post-fixed for 30 min in 2% paraformaldehyde before being transferred to PB. Brains were coded so investigators were blinded to genotype. Brains were sectioned into 50 µm slices in PB using a vibratome. Sections were then treated with 1% sodium borohydride in PB for 30 min to reduce free aldehydes, followed by extensive rinsing in PB. The tissue was subsequently transferred to 0.01 M tris-buffered saline (TBS), pH 7.6. Some sections were further processed by transferring them to a cryoprotectant solution and frozen for future use, while the remaining sections were immediately processed for immunogold-silver labeling of TH.

Blocking solution was prepared in TBS containing 3% goat serum, 1% BSA, and 0.04% Triton X-100, and applied to the tissue for 30 min to enhance antibody penetration. The primary antibody, mouse anti-TH (Chemicon), was diluted 1:5000 in the blocking solution and incubated with the tissue overnight at room temperature. After incubation, sections were washed in TBS, followed by 0.01 M phosphate-buffered saline (PBS), pH 7.4. They were then incubated for 30 min in a washing buffer consisting of PBS, 0.8% BSA, 0.1% fish gelatin, and 3% goat serum.

Next, sections were transferred to a solution containing 0.8 nm gold-conjugated anti-mouse secondary antibody, diluted 1:50 in the washing buffer, and incubated overnight at room temperature. The following day, the sections were rinsed thoroughly in washing buffer, followed by PBS, and incubated for 10 min in 2.5% glutaraldehyde in PBS to stabilize the gold particles. After rinsing in PBS, sections were treated with the proprietary Enhancement Conditioning Solution (ECS; Aurion/Electron Microscopy Sciences). The sections were then incubated in Aurion R-Gent SE-EM kit reagents at room temperature for an empirically determined time of approximately 90–120 min. After further rinsing in ECS, the sections were transferred to PB.

For electron microscopic visualization, immunogold-silver labeled tissue was subjected to osmication, dehydration, and plastic embedding. Sections were incubated in 2% osmium tetroxide in PB for 30 min, followed by rinsing in PB. The tissue was then sequentially dehydrated in 30%, 50%, 70%, and 95% ethanol solutions, and subsequently exposed twice to 100% ethanol for 10 min each, followed by two 10-min exposures to propylene oxide. The sections were incubated overnight in a 1:1 mixture of epoxy resin (Epon; EMBed-812, Electron Microscopy Sciences) and propylene oxide before being transferred to pure Epon for 2 h. The sections were embedded in Epon between sheets of commercial plastic and polymerized under heavy lead weights at 62 °C for 72 h.

The embedded tissue sections containing the dorsal striatum were mounted on plastic blocks, and excess tissue was trimmed, leaving a trapezoidal region within the dorsolateral striatum. This region of interest was then sectioned into ultrathin (50–60 nm) slices using an ultramicrotome and collected onto copper-400 mesh grids. The ultrathin sections were counterstained with uranyl acetate and lead citrate.

Ultrathin tissue sections were examined using a Morgagni FEI transmission electron microscope at 36000X magnification. Sampling of TH-immunolabeled profiles was done near the interface between tissue and epoxy resin, where maximal antibody penetration occurred. To decrease the collection of false positive results, TH-labeled axons were defined as profiles containing a minimum of three gold-silver particles, with many axons exhibiting considerably more than three particles.

For ultrastructural analysis, mitochondria were classified as healthy or unhealthy based on predefined morphological criteria. Healthy mitochondria exhibited intact cristae architecture, electron-dense matrix, and elongated or tubular morphology. Unhealthy mitochondria were identified by one or more of the following features: disrupted or fragmented cristae, mitochondrial swelling, vacuolization, reduced matrix electron density, or irregular morphology. Mitochondrial classification was performed by investigators blinded to genotype.

### Measurement of real-time oxygen consumption rate (OCR)

Real-time oxygen consumption rate (OCR) in acute brain slices was measured using a Seahorse XF24 analyzer (Seahorse Bioscience) with minor modifications to the manufacturer’s protocol. To optimize conditions for OCR measurement, various combinations of slice thickness (150 µm or 200 µm) and punch size (1.0 mm, 1.5 mm, and 2.0 mm) were tested. Brain slices were secured to the center of the mesh in an islet capture plate as described previously [26]. The microplate was then incubated at 37 °C for 1 h to equilibrate temperature and pH.

Drugs and inhibitors were diluted in prewarmed (37 °C) respiration buffer to stock concentrations (10×, 11×, 12×, and 13× the working concentrations for ports A, B, C, and D, respectively) and preloaded into the reagent ports of a sensor cartridge. The sensor cartridge had been hydrated overnight in Seahorse calibration solution at 37 °C without CO_2_. Each injection had a volume of 75 µL. Following a 30-min calibration and equilibration period, OCR was measured in each well of the plate using a program consisting of 3-min mixing, 3-min waiting, and 2-min measurement cycles. Baseline OCR was determined with four measurements, followed by sequential injections of port A (10 mM pyruvate, 3 X measurements), port B (10 μM oligomycin, 8 X measurements), port C (10 μM or other concentrations of FCCP, 5X measurements), and port D (20 μM antimycin A, 6 X measurements). Basal respiration was determined from OCR measurements obtained prior to oligomycin treatment. ATP-linked respiration was calculated as the oligomycin-sensitive component of OCR. Maximal respiration was determined from OCR measurements following FCCP treatment. Proton leak was calculated from OCR remaining after oligomycin treatment, and non-mitochondrial respiration was determined from OCR measurements following antimycin A treatment. The parameters for load, measure, and calibration distance of probe head were set to 27,800. Reagents used in Seahorse experiments included BSA (A6003), glucose (G8270), sodium pyruvate (P2256), L-glutamine (C3126), oligomycin from streptomyces diastatochromogenes (O4876), carbonyl cyanide 4-(trifluoromethoxy) phenylhydrazone (FCCP) (C2920), antimycin A from streptomyces sp. (A8674), which were purchased from Sigma Aldrich (St. Louis, MO) and rotenone (3616, Tocris). In addition, stainless steel biopsy punches were purchased from Miltex (York, PA), and XF24 Islet Flux-Paks from Seahorse Bioscience (Billerica, MA). Details of key resources and reagents are provided in Table 1.

**Table 1:** Key resources and reagents

Reagents or Resources	Source	Identifier
**Reagents**		
Aldrithiol	Sigma	Cat #143049
Bovine serum albumin (BSA)	Sigma	Cat #A6003
Dithiothreitol (DTT)	Sigma	Cat #D0632
Genipin	Sigma	Cat #G4796
Isradipine	Tocris	Cat #2004
MitoSOX Red	Invitrogen	Cat # M36008
N-(2-mercaptopropionyl)-glycine (MPG)	Sigma	Cat #M6635
Tetramethylrhodamine methyl ester (TMRM)	Invitrogen	Cat #T668
Antimycin A	Sigma	Cat #A8674
Carbonyl cyanide 4-(trifluoromethoxy) phenylhydrazone (FCCP)	Sigma	Cat #C2920
L-glutamine	Sigma	Cat #C3126
Oligomycin	Sigma	Cat #O4876
Rotenone	Tocris	Cat #3616
Sodium pyruvate	Sigma	Cat #P2256
**Resources**		
XF24 Islet capture microplate	Agilent	Cat #101122-100
XF24 Islet capture FluxPaks	Agilent	Cat #101174

### Detection of mitochondrial superoxide

As Santini described [27], acute coronal brain slices (100 μm) were prepared from mice expressing TH-GFP in dopaminergic neurons and incubated for 1.5 h at 35 °C in carbogenated aCSF. Slices were then transferred to a custom-made small chamber containing oxygenated aCSF supplemented with 5 μM MitoSOX Red mitochondrial superoxide indicator (Invitrogen) and incubated for 10 min. Immediately following dye loading, slices were fixed overnight at 4 °C in ice-cold 4% paraformaldehyde (PFA) in PBS. Fixed slices were mounted onto glass slides using a DAPI-containing mounting medium (Vector Laboratories).

Fluorescence imaging of the dorsal striatum and SNc was performed using a Zeiss LSM 900 confocal microscope. All acquisition parameters (pinhole, gain, offset, and contrast) were held constant across sections within the same experiment. Dopaminergic neurons were identified based on TH-GFP fluorescence, and regions of interest were defined for quantitative analysis. MitoSOX fluorescence integrated intensity in DA and non-DA neurons was quantified using a custom MATLAB-based script.

### Real-time PCR

Following the protocol described by Guzman et al., [5] total RNA from the SN region was extracted for quantification of UCP mRNA levels. (Primer sequences are provided in Table 2). Ventral midbrain regions, encompassing the SNc and VTA, were micro-dissected from 10-month-old LRRK2 WT and BAC-hR1441G mice. Total RNA was extracted using TRIzol reagent (Invitrogen), and cDNA synthesis was performed via reverse transcription (Quanta Biosciences) using oligo-(dT)15 primers.

**Table 2:** qPCR primer

Primer	Sense/anti-sense
mActin-ß	CATTGCTGACAGGATGCAGAAGG TGCTGGAAGGTGGACAGTGAGG
mUCP2	TAAAGGTCCGCTTCCAGGCTCA ACGGGCAACATTGGGAGAAGTC
mUCP4	CCTAAAGCTGTGGCAAGGAG CAATGACCGATTTCCAGAGG
mUCP5	GTGCTGCAATCGTTGTGGGAGT GCCAAACCACAGGTGAAACTGG

Quantitative PCR (qPCR) was carried out to analyze cDNA from each sample, employing SYBR Green as the fluorescent dye and sense/antisense primers synthesized by Integrated DNA Technologies. The qPCR cycling parameters included an initial denaturation step at 95 °C for 3 min, followed by 40 cycles of 15 sec at 94 °C, 1 min at 60 °C, and 30 sec at 72 °C. SYBR Green fluorescence was measured after each cycle.

Relative mRNA expression levels were calculated using the comparative C_T_ (ΔΔC_T_) method. Gene-specific mRNA expression was normalized to β-actin levels to obtain Δ*C*_T_ values (Δ*C*_T_ = *C*_T_(UCP4/5) − *C*_T_(ß-actin)). Paired Δ*C*_T_s for WT and BAC-hR1441G groups and the mRNA abundance were calculated from the equation 2 ^-∆∆ C^_T_. All samples were analyzed in triplicate to ensure accuracy.

### Western Blot

Mouse SN slices were homogenized in RIPA lysis buffer containing 1X protease inhibitor cocktail and centrifuged at 21,000 g for 30 min at 4 °C. Protein concentration was determined using the BCA protein assay kit. A total of 200 µg of protein was mixed with 4X Laemmli buffer and β-mercaptoethanol to achieve a final concentration of 1 µg/µl in 1X Laemmli buffer and 10% β-mercaptoethanol, then boiled for 5 min at 95 °C. A total of 10–20 µg of protein per lane was loaded onto 10% polyacrylamide gels and electrophoresed in Tris/glycine buffer, followed by transfer to 0.2 µm PVDF membranes. The PVDFs were blocked with 5% non-fat dry milk in TBS containing 0.05% Tween-20 (TBST) for 1–2 h at room temperature with gentle shaking at 20 rpm, and then incubated with primary antibodies overnight at 4 °C. After washing TBST three times, the PVDFs were incubated with the appropriate horseradish peroxidase (HRP)-conjugated secondary antibody for 1 h at room temperature with gentle shaking at 20 rpm. The PVDFs were then washed three times with TBST, developed using Femto Sensitivity Substrate, and imaged using a ChemiDoc MP Imager (Bio-Rad). Antibodies, protein assay kits, and buffer solutions are listed in Table 3.

**Table 3:** List of antibodies and Immunoblotting reagents

Antibody	Company	Source	Catalog	Dilution
MCU	CST	Rabbit	14997S	1:1000
MICU1	Sigma	Rabbit	HPA037480	1:1000
MICU2	Abcam	Rabbit	ab101465	1:1000
MICU3	Sigma	Rabbit	HPA024771	1:1000
UCP4	Santa Cruz	Mouse	sc-365295	1:500
HRP Goat Anti-Mouse IgG (H+L)	Jackson ImmunoResearch Laboratories, Inc		115–035-166	1:10000
HRP Goat Anti-Rabbit IgG (H+L)	Jackson ImmunoResearch Laboratories, Inc		111–035-144	1:10000
Goat Anti-Mouse IgG, IgM (H+L) Cross-Adsorbed Secondary Antibody, HRP	Invitrogen		31446	1:10000
SuperSignal™ West Femto Maximum Sensitivity Substrate	Thermo Scientific		34096	
Pierce™ BCA Protein Assay Kits	Thermo Scientific		23227	
Immun-Blot PVDF Membrane, Roll, 26 cm × 3.3 m	Bio-Rad		1620177	
RIPA Lysis and Extraction Buffer	Thermo Scientific		89900	
Pierce Protease and Phosphatase Inhibitor Mini Tablets, EDTA-Free	Thermo Scientific		A32961	

### Stereotaxic injection of AAV

Mice were anesthetized with isoflurane (1–2%) or ketamine/xylazine (100 mg/10 mg/kg). A small craniotomy, approximately 0.8 mm in diameter, was made over the right midbrain (−3.4 mm caudal, +1.0 mm lateral). To achieve expression of cyto-GCaMP6 or mito-GCaMP6 in DA neurons, 0.1–0.2 μl of AAV-TH-cyto-GCaMP6 or AAV-TH-mito-GCaMP6 virus (10^13 vg/ml, a gift from Dr. James Surmeier’s lab at Northwestern University) was pressure-injected through a syringe into the midbrain at a depth of −4.2 mm ventral from the dura surface. Following the injections, the incisional wound on the skull was sealed with tissue adhesive (3 M Vetbond). The mice were placed on a heating pad until fully recovered and returned to the isolation room. Carprofen (5 mg/kg, Sigma Aldrich) was administered intraperitoneally once per day for at least 3 days to reduce pain.

### Two-photon laser scanning microscopy (2PLSM) imaging of mitochondrial functions in brain slices

The brain slices were positioned within a recording chamber and perfused with oxygenated aCSF at a flow rate of 1.0 mL/min. Optical images of fluorescent signals were acquired using an Ultima multiphoton laser scanner (Bruker) for BX51/61WI, Olympus microscope with a Titanium-sapphire laser (Chameleon-Ultra2, Coherent Laser Group), equipped with a 20 × 1.0 NA water immersion objective (XLUMPLFL20XW, Olympus). The average laser power for imaging was less than 20 mW. Images were captured in 16-bit resolution and 4 μs dwell time, with regions of interest set at 512 × 512 pixels. All experiments were conducted at 34–35 °C.

### Mitochondrial roGFP (mito-roGFP) imaging

Acute brain slices expressing mitochondria-targeted roGFP were transferred to a recording chamber mounted on a two-photon microscope and continuously perfused with aCSF maintained at physiological temperature (34–35° C).

To calibrate the dynamic range of the roGFP sensor and determine the optimal excitation wavelengths for ratiometric imaging, brain slices were sequentially perfused with the reducing agent dithiothreitol (DTT, 2 mM; Sigma-Aldrich) for 45 min to achieve maximal reduction of roGFP, followed by the oxidizing agent aldrithiol (Ald, 100 μM; Sigma-Aldrich) for 45 min to induce maximal oxidation. Excitation spectra were collected under basal, fully reduced, and fully oxidized conditions. Based on these calibration experiments, 900 nm was identified as the optimal excitation wavelength for the reduced form of roGFP, whereas 800 nm preferentially excited the oxidized form, providing maximal separation between redox states (Extended Data Figs. 2–3). For all subsequent experiments, roGFP fluorescence was sequentially acquired at 800 nm and 900 nm excitation using a pixel size of 0.16–0.23 μm and a pixel dwell time of 4 μs. Emitted fluorescence (wavelength: 490-550 nm) was collected using a photomultiplier tube (PMT) detector. Mitochondrial redox status was quantified as the fluorescence intensity ratio obtained at 800 nm and 900 nm excitation wavelengths (F800/F900). An increase in the F800/F900 ratio indicates a more oxidized mitochondrial redox state, whereas a decrease in the ratio reflects a more reduced state. Laser power, PMT gain, and image acquisition parameters were maintained constant across experimental groups whenever feasible. Recordings with a drifting baseline were excluded from the analysis.

To investigate the effects of L-type calcium channel blockers, and LRRK2 kinase inhibitor on oxidative stress, brain slices were incubated in oxygenated aCSF containing isradipine (Tocris-Cookson, 10 µM) for 30 min, or GNE-7915 (3 µM), a kind gift from Dr. Nathanael Gray at Harvard Medical School, for 2 h before imaging [28]. Details of key resources and reagents are provided in Table 1.

### 2PLSM cyto-GCaMP6 and mito-GCaMP6 imaging

Mitochondrial Ca^2+^ levels were measured with the mitochondrially targeted Ca^2+^-sensitive probe mito-GCaMP6. Two to three weeks after AAV virus injection, mice were prepared for coronal brain slice sectioning following: transcardial perfusion with ice-cold, oxygenated cutting solution. After a recovery period of 30–45 min, the coronal brain slices were placed in a chamber perfused with oxygenated aCSF. To measure free Ca^2+^ levels in mitochondria, the slices were sequentially treated with the following solutions: (1) regular aCSF, (2) 500 µM EGTA + aCSF without Ca^2+^ for 30–40 min, (3) 500 µM EGTA + 1 µM ionomycin + aCSF without Ca^2+^ for 30 min, and (4) 1 µM ionomycin + aCSF with high Ca^2+^ (3 mM) for 20 min. Time series images were acquired every 5 min to determine the minimal and maximal fluorescence intensity. The fluorescence intensity at the end of treatment (2) was defined as the minimum fluorescence (F_min_), and the fluorescence intensity at the end of treatment (4) was defined as the maximum fluorescence (F_max_). The relative free calcium level was calculated using the equation: (F - F_min_)/(F_max_ - F_min_).

### Mitochondrial TMRM imaging

Tetramethylrhodamine methyl ester (TMRM; Invitrogen) was used to monitor mitochondrial membrane potential. A 10 mM TMRM stock solution prepared in DMSO was diluted in oxygenated aCSF to a final working concentration of 2-4 µM. Brain slices were incubated in the oxygenated TMRM-containing aCSF at 34–35 °C for 30 min to allow for dye equilibration and accumulation within the mitochondrial matrix. Following incubation, the slices were transferred to oxygenated TMRM-free aCSF and washed for 30 min to remove excess dye. Brain slices were put in the chamber under the microscope for imaging following aCSF solution perfusion at physiological temperatures (34–35 °C). Fluorescence (550–640 nm) was collected using an 850-nm excitation beam. Time-series scanning (300 frames) was performed with a dwell time of 4 μs per pixel at a scanning rate of 2–3 frames/s.

To investigate the effects of UCPs, calcium influx, and oxidative stress on MMP, slices were incubated in oxygenated aCSF containing genipin (10 μM, Sigma), isradipine (10 µM, Tocris-Cookson), or N-(2-mercaptopropionyl)-glycine (MPG, 10 µM, Sigma) for 30 min, respectively. Details of key resources and reagents are provided in Table 1.

### Image data analysis

Images were processed using open-source software Fiji (NIH) and commercial software MATLAB (Version 8.5.0 R2015a, Mathworks). Image registration was performed to correct for drift and ensure alignment across sequential image acquisitions.

#### roGFP analysis

DA terminals in the striatum and DA soma in the SNc area were segmented and selected as the regions of interest (ROIs). Mitochondrial oxidation level was quantified as the fluorescence intensity ratio obtained at 800 nm and 900 nm excitation wavelengths (F800/F900). An increase in the F800/F900 ratio indicates a more oxidized mitochondrial redox state, whereas a decrease reflects a more reduced state. Recordings exhibiting unstable baselines or excessive drift were excluded from analysis.

#### TMRM analysis

As described by Guzman [5], signals from TMRM and TH-GFP were acquired using an 850-nm excitation beam (80 MHz pulse repetition frequency, <250 fs pulse duration) in a fixed focal plane, DA neurons were identified based on TH-GFP fluorescence, and corresponding TMRM signals were analyzed within these cells (Extended data Fig. 12). Four to five ROIs were selected within each neuronal soma, and a nuclear ROI was used for background subtraction. TMRM fluorescence was background-corrected and monitored continuously over time. Recordings exhibiting excessive photobleaching or unstable baselines were excluded from further analysis.

Mitochondrial membrane potential (MMP) flickering events were identified from background-subtracted TMRM fluorescence traces as transient deviations from the baseline exceeding ±2 standard deviations and persisting for at least five consecutive frames. Flickering regions were selected as ROIs, and the frequency and amplitude of flicker events were quantified during 100-s recording epochs. Transient fluctuations lasting fewer than five consecutive frames were considered noise or imaging artifacts and were excluded from analysis. Drug effects were evaluated by comparing baseline recordings with recordings obtained 30–60 min after treatment.

### Digital spatial profiling

Formalin-fixed paraffin-embedded (FFPE) sections were processed and subjected to GeoMx RNA profiling (Nanostring Technologies, Seattle, WA, USA). ROIs/AOIs were defined using TH, GFAP, and Iba1 markers, and gene set enrichment analysis (GSEA) was performed to identify enriched pathways in LRRK2 mutant DA neurons. Further methodological details are provided below.

#### FFPE tissue preparation

Formalin-fixed paraffin-embedded (FFPE) tissues were prepared as follows: mice were anesthetized, and brains were fixed via transcardial perfusion with 10% neutral buffered formalin (Sigma-Aldrich). Brains were subsequently removed, post-fixed in the same formalin solution for 24 h at 4 °C and immersed in 70% ethanol for at least 1 h. Tissues were processed and embedded in paraffin using an automated tissue processor (Tissue-Tek VIP, Sakura Finetek USA Inc.) following the programmed protocol: 95% ethanol for 1h15m, 100% ethanol for 1h15m, 100% ethanol for 1h30m, 100% xylene for 1 h, 100% xylene for 1h30m, 100% xylene for 1h30m, paraffin for 1h30m at 58 °C, and paraffin for 2 h at 58 °C. FFPE tissues were then immersed in molten paraffin at 60 °C in a metal mold to form FFPE blocks. These blocks were cooled at 4 °C and stored at the same temperature until further use.

#### Tissue sectioning and slide preparation

The midbrains of FFPE mouse brains were coronally sectioned at 5 μm using a rotary microtome. Sections containing the SN and ventral tegmental area (VTA) were mounted on Leica BOND Plus microscope slides, with each slide holding 6 sections—3 from WT mice and 3 BAC-hR1441G mice. The slides were submitted to the Flow Cytometry and Human Immune Monitoring Core at Thomas Jefferson University for RNA profiling via the GeoMx RNA assay (Nanostring).

#### ROI and AOI selection

Tissue sections were stained with fluorescent morphology markers and nuclear counterstain for the identification of ROIs and areas of interest (AOIs). The morphology markers included an anti-TH antibody conjugated with Alexa 488 (dopaminergic neuron marker, Sigma #MAB5280X), an anti-GFAP antibody conjugated with Alexa 594 (astrocyte marker), and an anti-Iba1 antibody conjugated with Alexa 647 (microglia marker, Millipore #MABN92–AF647). The nucleus marker was Syto-13. For each tissue section, 2–3 ROIs were selected within the SN or VTA, and each ROI was subdivided into 1–3 AOIs representing TH+, GFAP+, or Iba1+ cell populations. Each AOI contained a minimum of 20 cells.

#### Quality control

Raw counts from GeoMx^TM^ Digital Spatial Profiling (DSP) were processed using GeoMx^TM^ Software. Out of 95 segments collected from 6 mice (3 harboring hLRRK2 R1441G mutations and 3 wild-type), 11 segments were excluded due to failure in quality control (QC) analysis. Following QC, 8021 out of 20,175 gene targets in the GeoMx Mouse Whole Transcriptome Atlas passed QC and were retained for downstream analyses.

#### Data normalization and analysis

To address systematic variability between AOIs, GeoMx^TM^ DSP raw counts were normalized to the 75th percentile of expression for each AOI, as described in the NanoString GeoMx^TM^ Data Analysis User Manual. Differential expression analysis was performed using a negative binomial model, implemented in the DESeq2 package in R.

#### Gene set enrichment analysis (GSEA)

GSEA was carried out to identify pathways dysregulated by the R1441G mutation in the SNc DA neurons compared with WT controls. GSEA was conducted using the ClusterProfiler package (version 4.12) in R, with gene sets ranging in size from 10 to 500 genes. Enrichment terms with *p*-values below 0.05 were considered significantly enriched. Additionally, GSEA was extended using gene set signatures from the Reactome pathway database and the KEGG dataset respectively, with normalized enrichment scores (NES) calculated to identify biological pathways linked to the R1441G mutation in DA neurons.

### Statistical analysis

Data are presented as mean ± standard deviation (SD) where applicable. Statistical analysis was performed with GraphPad Prism 8 (GraphPad software Inc.). Unpaired Mann-Whitney test, one-way ANOVA, and two-way ANOVA were applied, with a *p*-value of <0.05, considered statistically significant. If not specified, “N” refers to the number of animals used in each experiment, whereas “n” denotes the number of cells or brain slices analyzed.

## Results

### The LRRK2–R1441G mutation disrupts mitochondrial morphology and impairs respiratory function in striatal dopaminergic terminals

**Fig. 1:**
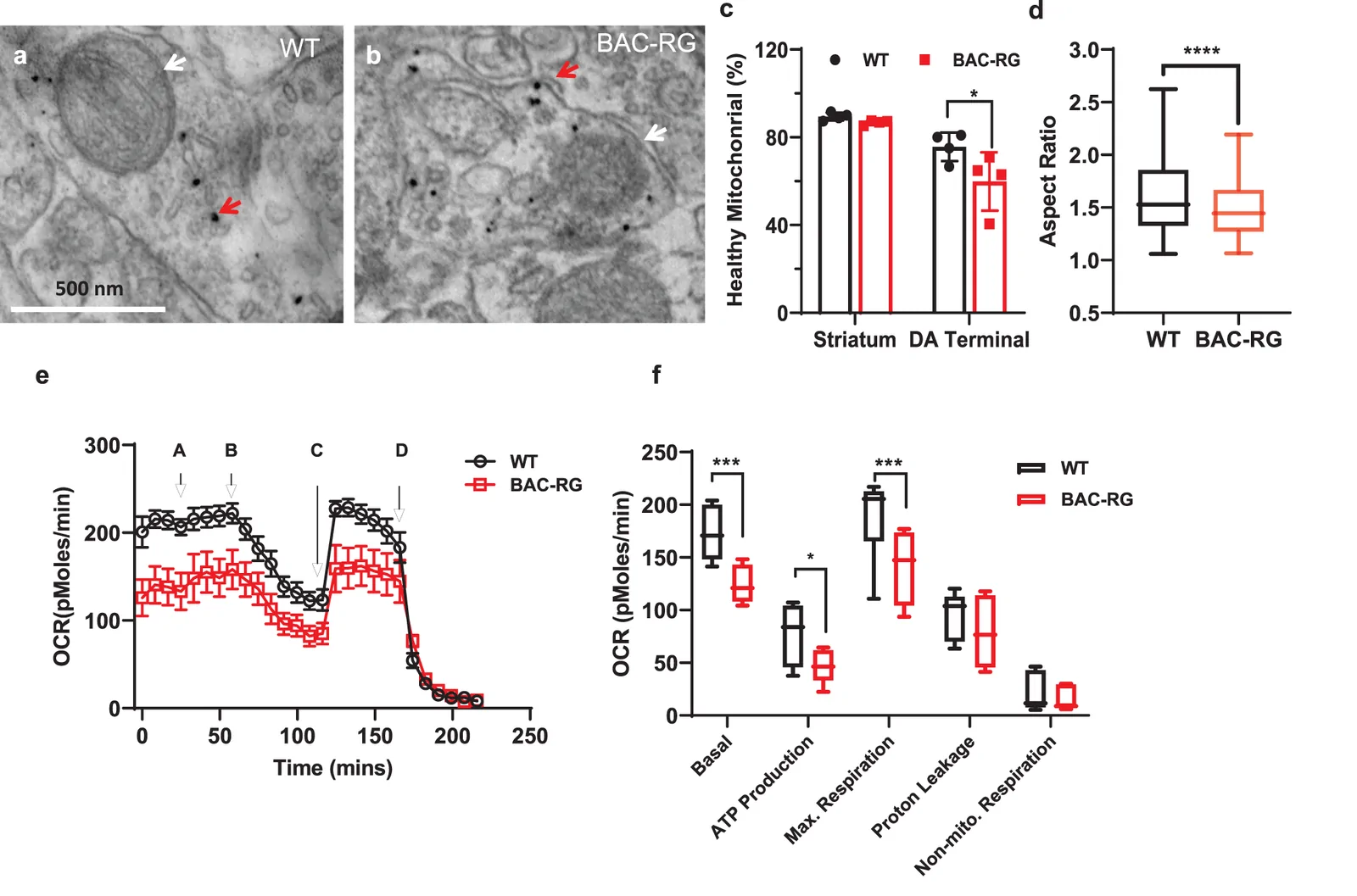
Mitochondrial morphology and respiration are impaired in the striatum of BAC-hR1441G mice. (**a, b**) Representative EM images of mitochondria following TH immunogold labeling in striatal sections from age-matched WT and BAC-hR1441G mice, respectively. Red arrows indicate TH-positive signals within DA terminals. White arrows indicate mitochondria located within or adjacent to DA terminals. (**c**) Quantification of mitochondrial morphology in the striatum and in DA terminals. BAC-hR1441G mice exhibited a significantly higher proportion of morphologically abnormal mitochondria in DA terminals compared with WT controls (striatum: WT, *N* = 4, *n* = 1300; BAC-RG, *n = 1231, p = 0.8499*; DA terminal: WT, *n* = 52; BAC-RG, *n* = 67, *p = 0.0225). (**d**) Quantification of mitochondrial aspect ratio. Mitochondria from BAC-hR1441G mice displayed significantly reduced aspect ratios compared with WT controls, indicating a more rounded mitochondrial morphology (Mann–Whitney test, ****p < 0.0001). (**e**) Seahorse analysis of mitochondrial respiration in acute striatal slices. *N *= 3 for each genotype. Slices were sequentially treated with 10 mM pyruvate (**A**), 10 μM oligomycin (**B**), 10 μM FCCP (**C**), and 20 μM antimycin A (**D**), and oxygen consumption rate (OCR) was measured in real time. (**f**) Quantification of Seahorse-derived respiratory parameters. BAC-hR1441G mice exhibited significantly reduced basal respiration, ATP-linked respiration, and maximal respiratory capacity, whereas proton leak and non-mitochondrial respiration were unchanged (Two-way ANOVA test, basal, WT vs BAC-RG, ***p = 0.0006; ATP production, WT vs BAC-RG, *p = 0.0478; Max. Respiration, WT vs BAC-RG, ***p = 0.0006; proton leakage, *p* = 0.5808; non-mitochondrial respiration, *p* = 0.9972).

Mutations in *LRRK2* represent the most common genetic cause of both familial and sporadic Parkinson’s disease (PD). Li et al. reported age-dependent DA release and motor deficits in BAC-hR1441G mice, implicating this mutation in PD pathogenesis [24]. Mitochondrial dysfunction is a key driver of DA neuron degeneration in the SNc, and accumulating evidence indicates that degeneration follows a ‘dying-back’ pattern, with early loss of striatal DA terminals preceding neuronal cell body degeneration [3, 29, 30]. To determine whether mitochondrial abnormalities occur within DA terminals, we combined TH immunolabeling with electron microscopy (EM) to examine mitochondrial ultrastructure and morphology in striatal DA terminals. In 10-month-old BAC-hR1441G mice, mitochondria in TH-positive terminals exhibited abnormal structural features, including cristae loss and irregular oval-shaped morphology (Fig. 1a, b). In contrast, WT mice displayed predominantly healthy mitochondria with regular tubular morphology in TH-positive terminals (Fig. 1c). Quantitative analysis of mitochondrial aspect ratio (i.e., major axis length divided by minor axis length) further revealed that mitochondria in BAC-hR1441G mice were closer to unity, suggesting increased mitochondrial fission (Fig. 1d). Despite these qualitative changes, the overall proportion of morphologically healthy mitochondria in the striatum did not differ significantly between BAC-hR1441G and WT mice. These findings indicate that the R1441G mutation leads to abnormal mitochondrial morphology only in DA terminals. Given the critical role of mitochondria in supporting synaptic energy demands and neurotransmission, these alterations may contribute to the early vulnerability of nigrostriatal dopaminergic projections in PD.

To determine whether these structural abnormalities are accompanied by bioenergetic deficits, we next assessed mitochondrial respiratory function using Seahorse extracellular flux analysis in acutely prepared striatal slices. Mitochondrial respiration was assessed following sequential administration of pyruvate (10 mM), oligomycin (10 μM), FCCP (10 μM), and antimycin A (20 μM) [26]. In 10-month-old BAC-hR1441G mice, we observed a significant reduction in basal OCR, ATP production, and maximal respiratory capacity (Fig. 1e, f; Extended Data Fig. 1). However, no significant differences in mitochondrial respiration were detected in younger (3- and 6-month-old) animals, suggesting a progressive decline in mitochondrial function with aging.

### The LRRK2–R1441G mutation induces age-dependent mitochondrial oxidative stress in SNc dopaminergic neurons and striatal terminals

The autonomous pacemaking activity of SNc dopaminergic neurons is critical for maintaining DA release and normal striatal function. This sustained electrical activity imposes substantial bioenergetic demands that are largely supported by mitochondrial oxidative phosphorylation. While oxidative phosphorylation efficiently generates ATP, it also produces reactive oxygen species (ROS), rendering SNc dopaminergic neurons particularly vulnerable to mitochondrial oxidative stress.

**Fig. 2:**
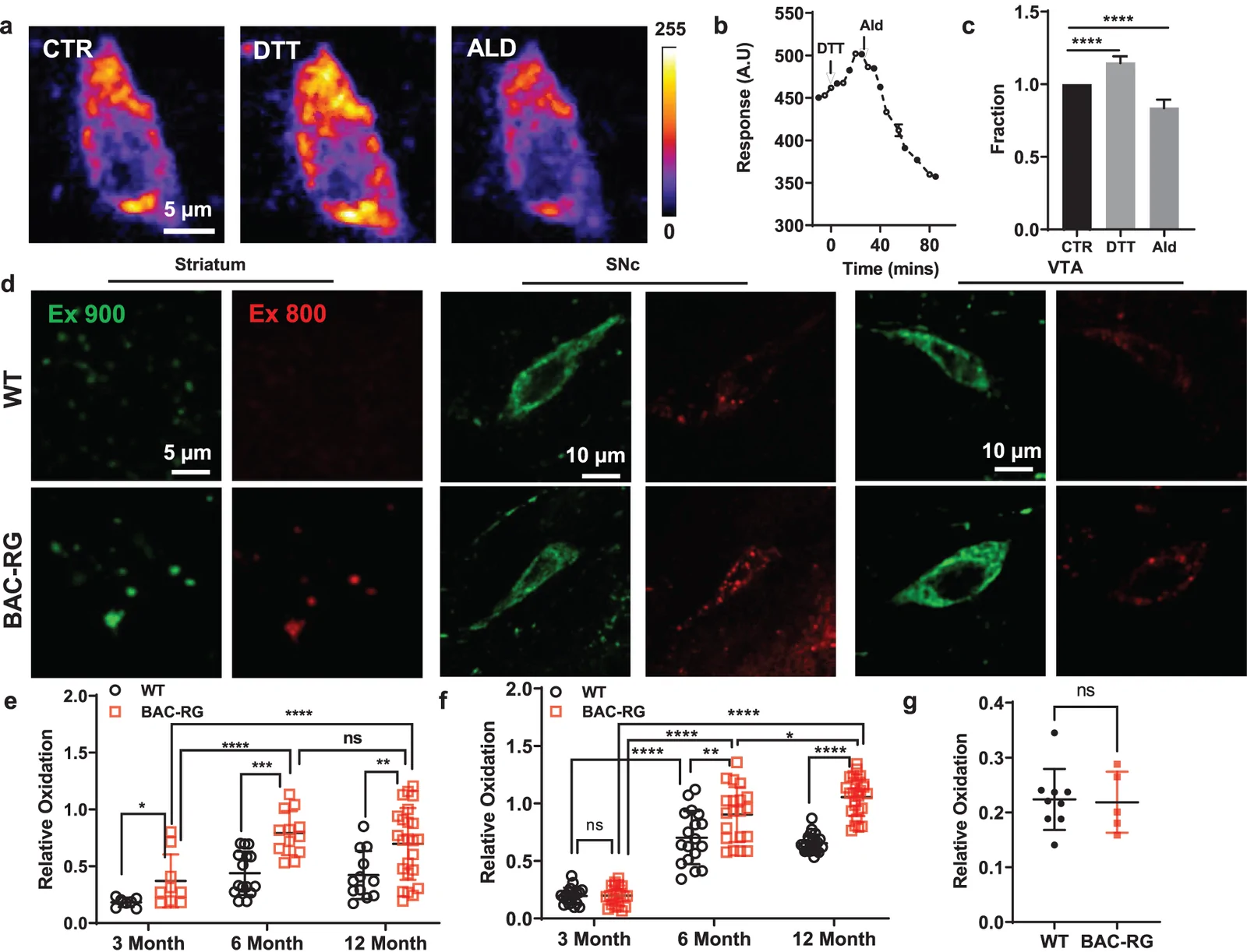
Age-dependent mitochondrial oxidative stress in BAC-hR1441G DA neurons. (**a**) Representative images of mito-roGFP-expressing SNc DA neurons under basal conditions and following treatment with dithiothreitol (DTT) or aldrithiol (ald). (**b**) Time course of mito-roGFP fluorescence during sequential DTT and ald treatment. (**c**) Quantification of mito-roGFP responses to DTT and ald treatment (*N* = 4, *n* = 6, Wilcoxon matched pairs signed rank test; CTR vs DTT, *p = 0.0313; CTR vs ald, *p = 0.0313). (**d**) Representative ratiometric mito-roGFP images acquired at 900 nm and 800 nm excitation wavelengths in DA neurons and terminals from the dorsal striatum (dSTR), SNc, and ventral tegmental area (VTA) of 6-month-old WT and BAC-hR1441G mice. (**e**) Quantification of mitochondrial oxidative stress in dSTR DA terminals. Oxidative stress levels showed an age-dependent increase, with BAC-RG mice demonstrating elevated oxidative stress at 3 months (*p = 0.0140), 6 months (***p = 0.0008) and 12 months (**p = 0.0051) compared to WT controls (3 pairs for 3-month-old WT, *n* = 10; BAC-RG, *n* = 7; 4 pairs for 6-month-old WT, *n* = 11; BAC-RG, *n* = 8; 3 pairs for 12-month-old WT, *n* = 12; BAC-RG, *n* = 20). (**f**) Quantification of mitochondrial oxidative stress in SNc DA neurons. Oxidative stress levels in SNc DA neurons increased with age, with BAC-hR1441G mice exhibiting significantly higher oxidative stress at 6 months (**p = 0.005) and 12 months (****p < 0.0001) compared to WT controls (3 pairs; DA neurons: 3-month-old WT, *n* = 18; BAC-RG, *n* = 21; 6-month-old WT, *n* = 18; BAC-RG, *n* = 21; 12-month-old WT, *n* = 24; BAC-RG, *n* = 25). (**g**) No significant difference in oxidative stress was observed in VTA DA neurons between 6-month-old WT and BAC-RG mice (3 pairs; DA neurons: WT, *n* = 9; BAC-RG, *n* = 5; Mann-Whitney test, *p* = 0.8981)

To determine whether mitochondrial oxidative stress is altered in the SNc and striatum of BAC-hR1441G mice, we crossed these animals with TH-mito-roGFP reporter mice, which express a mitochondria-targeted redox-sensitive green fluorescent protein (roGFP) under the control of the TH promoter. This strategy enabled cell type-specific monitoring of mitochondrial redox status in dopaminergic neurons and generated BAC-hR1441G/TH-mito-roGFP mice for subsequent imaging studies. Using two-photon laser scanning microscopy (2PLSM) combined with a ratiometric imaging approach (Fig. 2a–c; Extended Data Figs. 2–4), we assessed mitochondrial redox states in dopaminergic neurons in acute brain slices. BAC-hR1441G mice exhibited significantly increased mitochondrial oxidation levels in the SNc and dorsal striatum, particularly at 6 and 12 months of age (Fig. 2d–f; Extended Data Figs. 5–6). Notably, mitochondrial oxidative stress was already significantly elevated in striatal dopaminergic terminals of 3-month-old BAC-hR1441G mice, suggesting that mitochondrial dysfunction emerges early at presynaptic sites. This oxidative stress increase exhibited a distinct age-dependent pattern. In SNc DA neurons, oxidation levels progressively increased from 3 to 6 months and further at 12 months. In contrast, in striatal terminals, oxidative stress rose significantly between 3 and 6 months but plateaued thereafter, with no further increase between 6 and 12 months in BAC-hR1441G mice.

To determine whether the observed increase in mitochondrial oxidative stress was attributable to the R1441G mutation rather than overexpression of human LRRK2, we examined age-matched BAC-hLRRK2-WT mice. No significant differences in mitochondrial oxidative stress were detected between BAC-hLRRK2-WT mice and non-transgenic controls at approximately 7 months of age (Extended Data Fig. 7), indicating that overexpression of WT human LRRK2 alone is insufficient to induce elevated mitochondrial oxidative stress. To assess the contribution of LRRK2 kinase activity, acute brain slices were treated with the selective LRRK2 kinase inhibitor GNE-7915 (3 μM). Pharmacological inhibition of LRRK2 kinase activity significantly reduced mitochondrial oxidative stress in both striatal DA terminals and SNc DA neuron somata of BAC-hR1441G mice (Extended Data Fig. 8). These findings indicate that the elevated mitochondrial oxidative stress observed in BAC-hR1441G mice is attributable to the pathogenic R1441G mutation and is at least partially dependent on aberrant LRRK2 kinase activity. No significant differences in mitochondrial redox status were detected in VTA DA neurons between 6-month-old WT and BAC-hR1441G mice (Fig. 2g; Extended Data Fig. 9). To further assess the cellular specificity of this phenotype, we quantified mitochondrial superoxide levels using MitoSOX staining in TH-negative cells (non-DA cells) within the dorsal striatum and substantia nigra. No significant differences in MitoSOX fluorescence intensity of non-DA cells were observed between WT and BAC-hR1441G mice in either region (Extended Data Figs. 10–11).

Together, these findings demonstrate that the LRRK2-hR1441G mutation induces region-specific and age-dependent increases in mitochondrial oxidative stress, particularly in the SNc and dorsal striatum, but not in the VTA. This suggests that SNc DA neurons and their striatal projections are more vulnerable to oxidative stress, which may contribute to their selective degeneration in PD.

### Cytosolic and mitochondrial Ca^2+^ levels are elevated in BAC-hR1441G mice

**Fig. 3:**
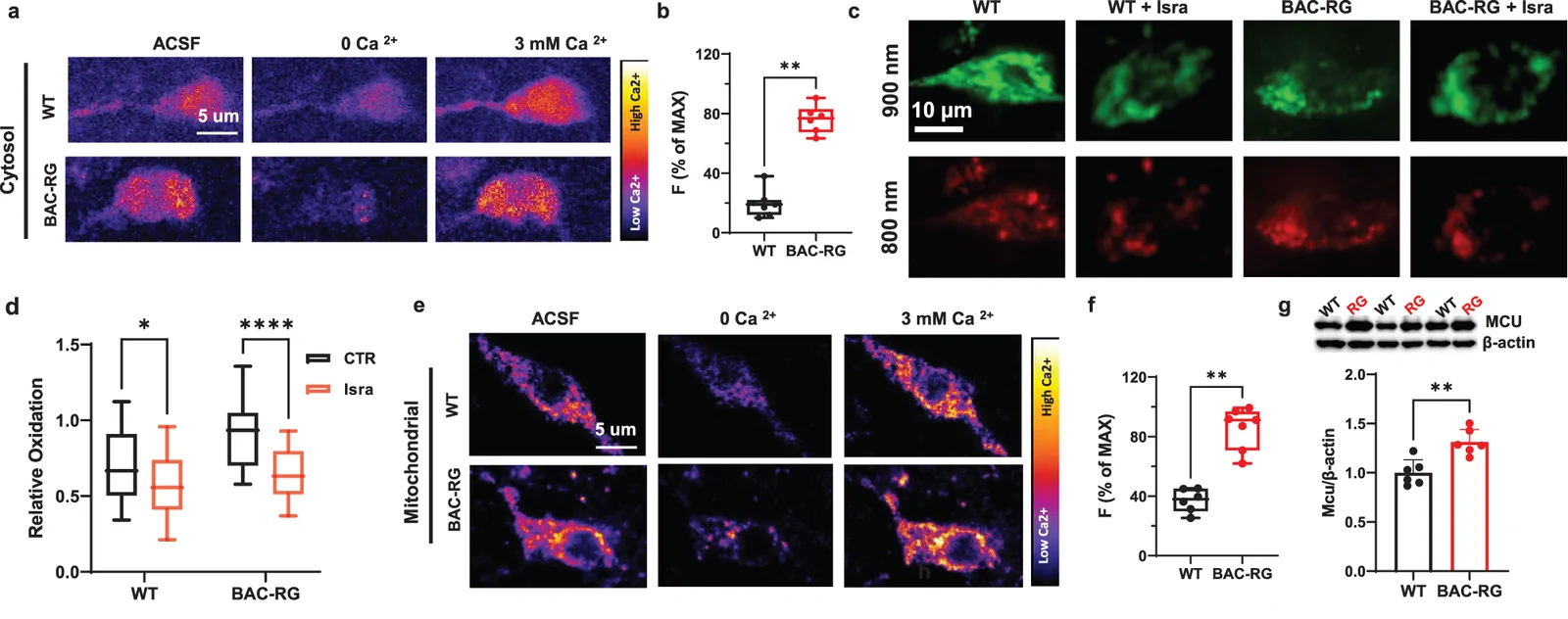
Elevated cytosolic and mitochondrial Ca^2 +^ levels in SNc DA neurons of 5-month-old BAC-hR1441G mice. (**a**) Pseudo-colored representative images of cytosolic calcium levels in SNc DA neurons from WT and BAC-hR1441G mice under three perfusion conditions: regular aCSF, aCSF with 0 mM Ca^2 +^ + EGTA + 1 μM ionomycin, and aCSF with 3 mM Ca^2 +^ + 1 μM ionomycin. (**b**) Quantification of cytosolic calcium levels revealed that the R1441G mutation significantly increased cytosolic calcium levels compared to WT mice (Mann-Whitney test, **p = 0.0012; WT: *N* = 3, *n* = 7; BAC-RG: *N* = 3, *n* = 6). (**c**) Representative images showing the effects of isradipine treatment on mitochondrial redox status in SNc DA neurons from WT and BAC-hR1441G mice. (**d**) Isradipine treatment significantly reduced redox levels in SNc DA neurons (Two-way ANOVA, WT vs WT + isra, *p = 0.0427, BAC-RG vs BAC-RG + isra, ****p < 0.0001). (**e**) Pseudo-colored representative images of mitochondrial calcium levels in WT and BAC-RG mice under three perfusion conditions: regular aCSF, aCSF with 0 mM Ca^2 +^ + EGTA + 1 μM ionomycin, and aCSF with 3 mM Ca^2 +^ + 1 μM ionomycin. (**f**) Quantification of mitochondrial calcium levels revealed a significant increase in mitochondrial calcium in BAC-hR1441G mice (Mann-Whitney test, **p = 0.0012; WT, *N* = 3 *n* = 6; BAC-RG, *N* = 4, *n* = 7). (**g**) Top: representative Western blot analysis of MCU expression in the SN of WT and BAC-RG mice. Bottom: quantification demonstrating significantly elevated MCU protein levels in BAC-hR1441G mice compared with WT controls (3 pairs, Mann-Whitney test, *p = 0.0138)

To investigate whether the elevated mitochondrial oxidative stress in BAC-hR1441G mice results from altered calcium homeostasis, we injected AAV-TH-cyto-GCaMP6 under the control of the TH promoter into the SNc to selectively target DA neurons. Quantification of cytosolic calcium levels revealed a significant increase in BAC-hR1441G mice, with normalized GCaMP6 fluorescence increasing from 19.71% in WT mice to 75.75% in BAC-hR1441G mice, indicating a substantial elevation in cytosolic calcium induced by the R1441G mutation (Fig. 3a–b).

To determine whether L-type calcium channel activity contributes to mitochondrial oxidative stress, we administered isradipine, a selective L-type calcium channel inhibitor. Isradipine significantly reduced mitochondrial oxidation in both WT and BAC-hR1441G DA neurons, with a more pronounced effect in BAC-hR1441G mice (Fig. 3c–d). These findings suggest that calcium influx through L-type calcium channels contributes to mitochondrial redox imbalance.

To further assess mitochondrial calcium levels, we injected AAV vectors expressing a mitochondria-targeted calcium indicator under the TH-mito-matrix promoter into the SNc of WT and BAC-hR1441G mice. The R1441G mutation significantly increased mitochondrial calcium levels (Fig. 3e–f). Western blot analysis further revealed upregulation of MCU, a key regulator of calcium influx into the mitochondria, in BAC-hR1441G mice, supporting a role for MCU-mediated calcium overload in mitochondrial dysfunction (Fig. 3g).

Collectively, these results demonstrate that calcium overload, driven by increased mitochondrial calcium uptake and elevated MCU expression, contributes to excessive ROS production in BAC-hR1441G DA neurons, establishing a mechanistic link between calcium dysregulation and oxidative stress.

### Mitochondrial membrane potential flickering reflects oxidative stress and is attenuated in BAC-hR1441G dopaminergic neurons

**Fig. 4:**
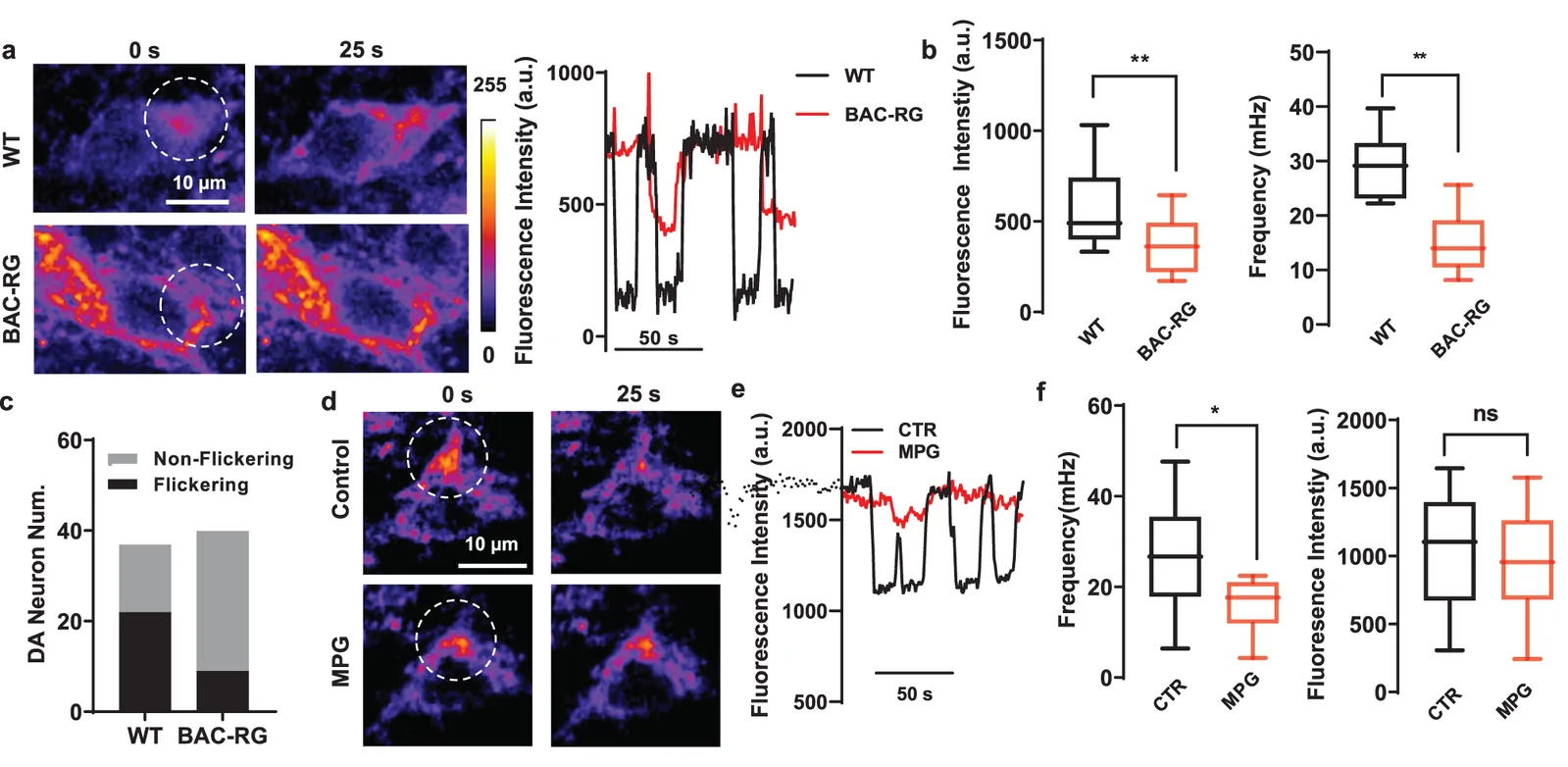
Attenuated mitochondrial membrane potential (MMP) flickering in SNc DA neurons of 6-month-old BAC-hR1441G mice (**a**) Left, representative TMRM images of mitochondria in SNc DA neurons from 6-month-old WT and BAC-hR1441G mice acquired at 0 and 25 s. Right, representative TMRM fluorescence traces from the indicated region of interest (ROI; white circle), illustrating differences in MMP dynamics between WT and BAC-hR1441G mice. (**b**) Left, quantification of MMP flickering amplitude showing a significant reduction in BAC-hR1441G mice compared with WT controls (Mann-Whitney test, **p = 0.0073). Right, quantification of MMP flickering frequency showing a significant reduction in BAC-hR1441G mice compared with WT controls (Mann-Whitney test, **p = 0.0011). (**c**) Quantification of SNc DA neurons exhibiting detectable MMP flickering events, demonstrating a reduced proportion of flickering neurons in BAC-hR1441G mice. (**d**) Representative TMRM images of SNc DA neurons before and after bath application of the antioxidant N-(2-mercaptopropionyl)-glycine (MPG). (**e**) Representative TMRM fluorescence traces before and after MPG treatment, showing reduced MMP flickering following MPG treatment. (**f**) Quantification of MMP flickering frequency (left) and amplitude (right) before and after MPG treatment (*N* = 3, *n* = 9). MPG significantly reduced MMP flickering events associated with oxidative stress (Mann-Whitney test, *p = 0.0260)

The mitochondrial membrane potential (MMP) plays a critical role in regulating ATP production and ROS generation [5, 24]. To investigate whether elevated oxidative stress in BAC-hR1441G mice is associated with alterations in MMP, we employed tetramethylrhodamine methyl ester (TMRM, 2–4 µM), a dye that selectively accumulates within mitochondria to measure MMP. Since TMRM labeling is not cell-type specific, we crossed BAC-hR1441G mice with TH-GFP reporter mice to selectively identify DA neurons among TMRM-labeled populations (Extended Data Fig. 12). In both WT and BAC-hR1441G mice at 6 months of age, DA neurons exhibited periodic oscillations in TMRM fluorescence, indicating transient depolarization and repolarization events of the MMP (Fig. 4a; Extended Data Video 1). The frequency, amplitude, and overall occurrence of MMP transients were significantly reduced in SNc DA neurons from BAC-hR1441G mice compared to WT controls (Fig. 4b, c). Notably, MMP flickering events, reflecting transient cycles of mitochondrial depolarization and repolarization, were more prominent in WT DA neurons than in BAC-hR1441G DA neurons (Fig. 4c). The frequency of MMP transients exhibited an age-dependent decline, decreasing progressively from 3 to 6 to 12 months in both genotypes (Extended Data Fig. 13a). BAC-hR1441G mice showed significantly reduced MMP transient frequencies at 6 and 12 months compared to WT mice, with no significant differences observed at 3 months (Extended Data Fig. 13b).

To determine whether oxidative stress contributes to MMP flickering, we treated brain slices with N-(2-mercaptopropionyl) glycine (MPG), a cell-permeable antioxidant. MPG treatment reduced MMP flickering frequency and decreased MMP amplitudes (Fig. 4d–f; Extended Data Video 2).

Collectively, these findings suggest that MMP flickering is associated with oxidative stress and that the R1441G mutation impairs MMP homeostasis in an age- and oxidative stress-dependent manner.

### Reduced UCP expression is associated with attenuated MMP flickering in BAC-hR1441G DA neurons

Previous studies have demonstrated that MMP flickering in SNc DA neurons reflects transient UCP-mediated mitochondrial uncoupling and is regulated by calcium influx through L-type Ca^2 +^ channels [22]. To confirm that MMP flickering observed in our system is regulated by these established mechanisms, we performed pharmacological manipulations targeting calcium entry and mitochondrial uncoupling. Inhibition of L-type Ca^2 +^ channels with isradipine significantly reduced the frequency and amplitude of MMP transients (Extended Data Fig. 14a-d; Extended Data Video 3). Similarly, the blockade of UCP activity with genipin attenuated MMP flickering events (Extended Data Fig. 14e–h; Extended Data Video 4). These findings confirm that the MMP flickering observed in our recordings is regulated by calcium- and UCP-dependent mechanisms previously described in SNc DA neurons.

**Fig. 5:**
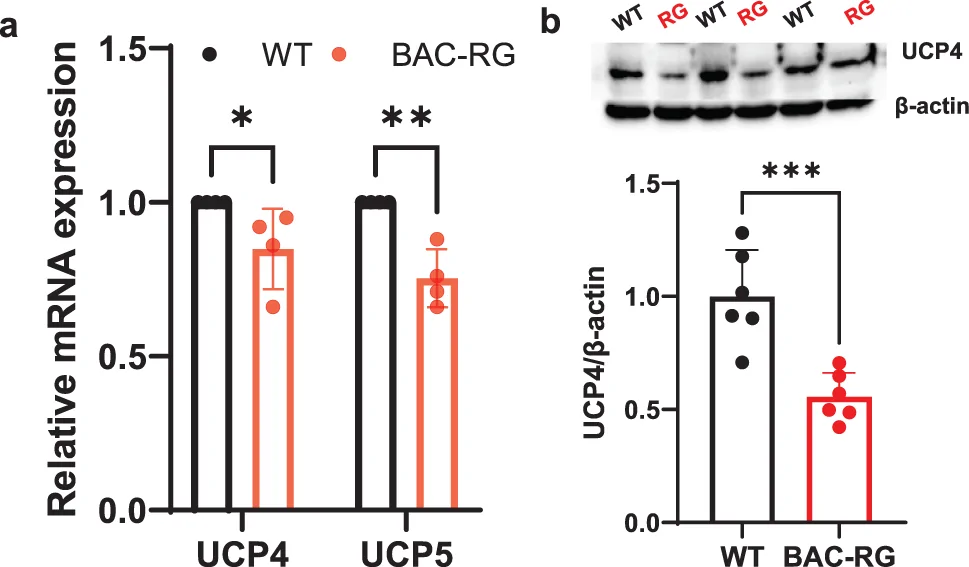
Reduced UCP expression in SNc DA neurons of BAC-hR1441G mice (**a**) Quantification of UCP mRNA expression in SNc DA neurons. BAC-hR1441G mice exhibited significantly reduced expression of UCP4 and UCP5 compared with WT controls (4 pairs, two-way ANOVA, WT vs BAC-RG, UCP4, *p = 0.0397, UCP5, **p = 0.0019). (**b**) Top, representative Western blot showing UCP4 expression in the SN. Bottom, quantification of UCP4 protein levels demonstrating a significant reduction in BAC-hR1441G mice compared with WT controls (4 pairs; Mann-Whitney test, **p = 0.0022)

UCPs are ion channels in the inner mitochondrial membrane that mitigate oxidative stress by dissipating the proton gradient across the membrane, thereby limiting excessive ROS production [5, 25]. Because BAC-hR1441G DA neurons exhibited attenuated MMP flickering despite elevated mitochondrial oxidative stress, we next examined whether UCP expression was altered by the R1441G mutation. Using microdissection of SNc DA neurons, we found significantly reduced mRNA levels of UCP4 and UCP5 in BAC-hR1441G mice compared with WT controls (Fig. 5a). Consistent with these findings, Western blot analysis of SN tissue revealed protein expression in BAC-hR1441G mice (Fig. 5b).

Together, these findings demonstrate that the R1441G mutation is associated with reduced UCP expression and attenuated UCP-mediated mitochondrial uncoupling. Given the established role of UCP-dependent uncoupling in limiting ROS production, impairment of this protective pathway may contribute to the elevated mitochondrial oxidative stress observed in BAC-hR1441G DA neurons.

### Digital spatial profiling identifies transcriptional signatures associated with mitochondrial dysfunction and oxidative stress in BAC-hR1441G SNc DA neurons

**Fig. 6:**
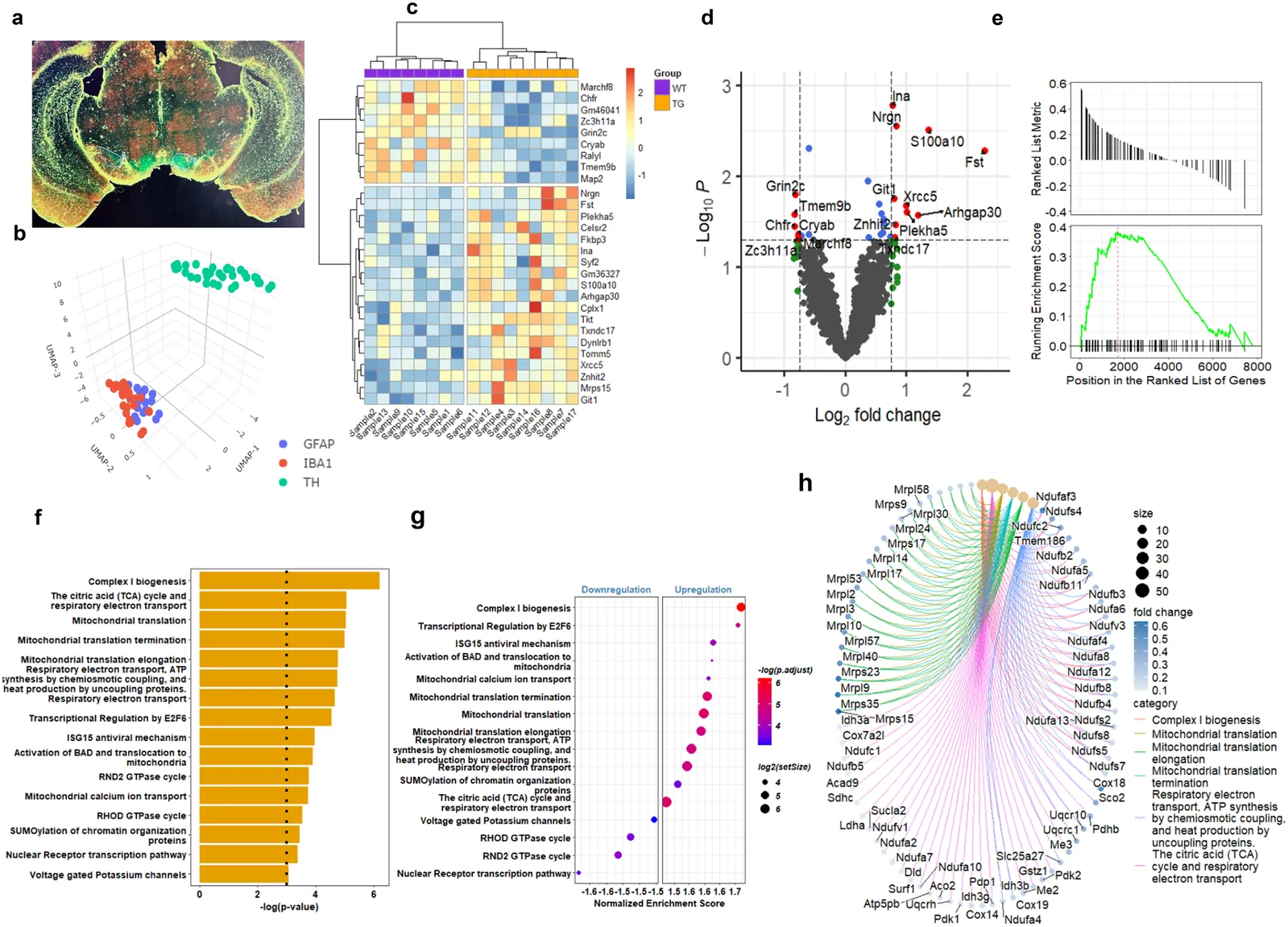
Digital spatial profiling reveals mitochondrial and redox pathway dysregulation in BAC-hR1441G SNc DA neurons. (**a**) Representative coronal sections showing TH, GFAP, and IBA1 labeling used for GeoMx digital spatial profiling of the SNc. (**b**) UMAP analysis demonstrating transcriptional segregation of DA neurons, astrocytes, and microglia. (**c**) Heatmap of significantly differentially expressed genes (DEGs) in SNc DA neurons from BAC-hR1441G and WT mice. (**d**) Volcano plot showing DEGs in SNc DA neurons between BAC-hR1441G and WT mice, with Log_2_ Fold changes plotted against -Log_10_ of the adjusted *p*-value for each gene. (**e**) Enrichment plot showing upregulation of Reactome pathways related to respiratory electron transport, ATP synthesis via chemiosmotic coupling, and heat production by uncoupling proteins in DA neurons from BAC-hR1441G mice relative to WT controls. (**f**) Top 16 significantly enriched Reactome pathways identified from DEGs in SNc DA neurons, ranked by statistical significance. (**g**) Gene set enrichment analysis (GSEA) in SNc DA neurons is presented as a hierarchically clustered heatmap. The color key represents the Log10 *p*-value for the enrichment score, while the circle size reflects the number of leading genes associated with each enrichment term. (**h**) CNETplot visualizing the complex associations between genes and significantly enriched Reactome terms in DA neurons. Node size corresponds to the statistical significance (adjusted *p*-value) of the term, while gene node colors indicate their Fold change values. Edges denote the relationship between genes and their associated enrichment terms

To elucidate the molecular pathways disrupted by the R1441G mutation, we performed GeoMx digital spatial profiler (DSP) analysis on SNc DA neurons from BAC-hR1441G and WT mice and compared their transcriptional profiles. Uniform Manifold Approximation and Projection (UMAP) analysis revealed distinct clustering of SNc DA neurons, microglia, and astrocytes, indicating clear transcriptional separation among these cell populations (Fig. 6a–b). The analysis identified 19 significantly upregulated and 9 downregulated genes in mutant DA neurons (Fig. 6c). Volcano plots further highlighted the differentially expressed genes (DEGs) associated with the R1441G mutation (Fig. 6d).

Functional enrichment analysis of DEGs revealed significant alterations of pathways related to respiratory electron transport, and UCP-associated thermogenic processes in BAC-hR1441G DA neurons (Fig. 6e). Among the top 16 significantly enriched pathways identified using Reactome (Fig. 6f–g), the majority were associated with redox-related mitochondrial processes, including “Complex I biogenesis” and “The citric acid (TCA) cycle and respiratory electron transport”. Consistent with these findings, pathway analysis using the KEGG dataset revealed similar enrichment, particularly in oxidative metabolism pathways such as “Oxidative phosphorylation – Mus musculus” (Extended Data Fig. 15a-c). Notably, many of the DEGs contributing to pathway enrichment were linked to mitochondrial respiration, calcium homeostasis, and redox-related processes. These transcriptomic findings provide independent support for the mitochondrial abnormalities and oxidative stress phenotypes observed in BAC-hR1441G DA neurons.

**Fig. 7:**
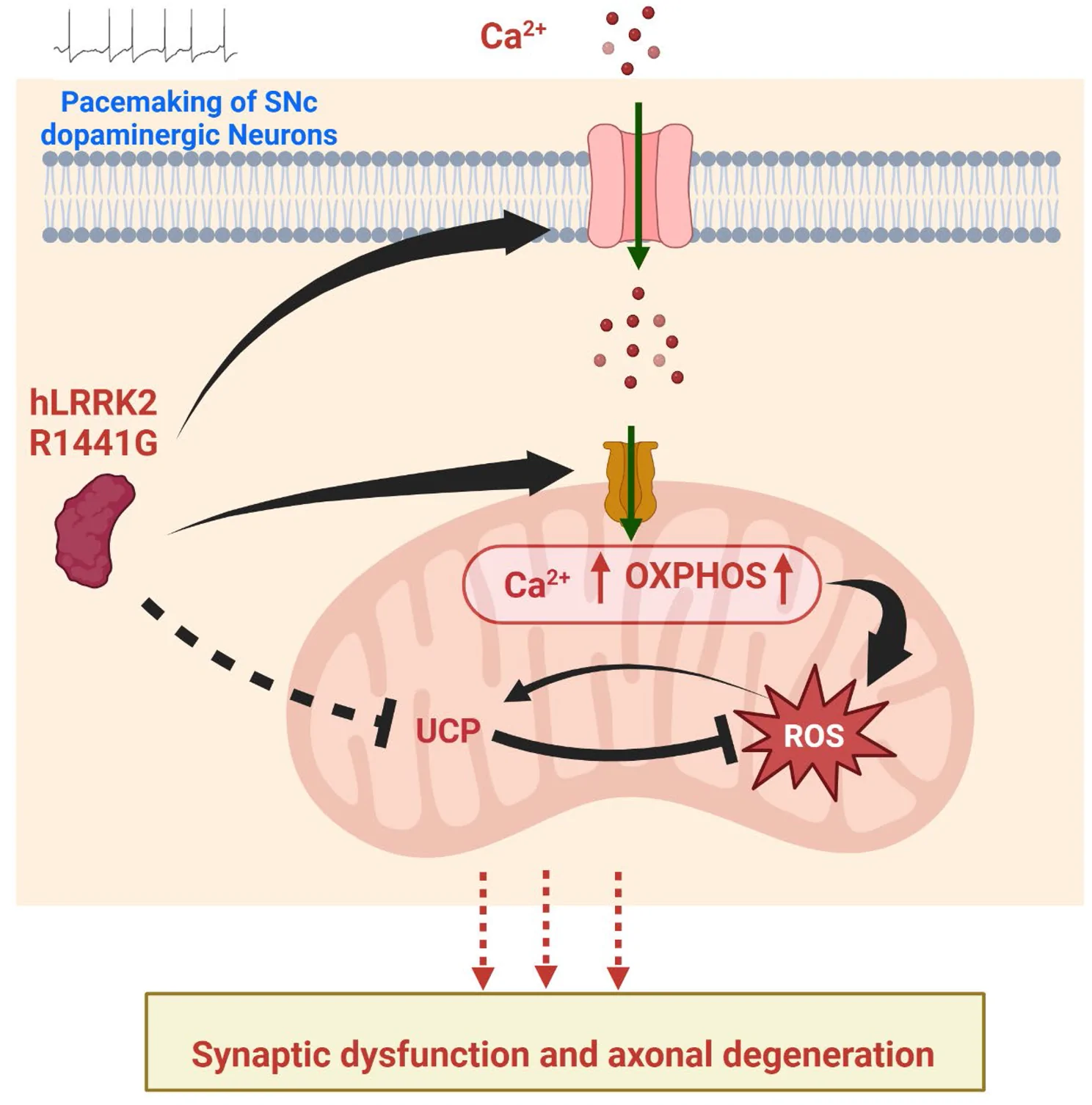
Proposed model linking the R1441G mutation to mitochondrial oxidative stress in SNc DA neurons. The R1441G mutation disrupts mitochondrial calcium and redox homeostasis in SNc DA neurons. Increased cytosolic and mitochondrial Ca^2 +^ levels, accompanied by elevated MCU expression, promote mitochondrial calcium accumulation and are associated with increased mitochondrial oxidative stress. In parallel, the R1441G mutation reduces UCP4 and UCP5 expression and attenuates UCP-mediated MMP flickering, a protective mechanism that normally limits excessive ROS generation through mild mitochondrial uncoupling. Together, these alterations shift the balance toward increased mitochondrial ROS production and oxidative stress, thereby contributing to mitochondrial dysfunction in vulnerable nigrostriatal DA neurons and promoting the synaptic and axonal deficits associated with the R1441G mutation. These findings highlight a critical role for the R1441G mutation in disrupting mitochondrial homeostasis and identify mitochondrial calcium dysregulation and impaired UCP-dependent uncoupling as potential mechanisms contributing to neurodegeneration in Parkinson’s disease

In summary, our imaging, physiological, biochemical, and spatial transcriptomic analyses support a model in which the R1441G mutation disrupts mitochondrial homeostasis in vulnerable SNc DA neurons (Fig. 7). BAC-hR1441G mice exhibit elevated cytosolic and mitochondrial Ca^2+^ levels, increased mitochondrial oxidative stress, impaired MMP dynamics, and reduced expression of the neuronal uncoupling proteins UCP4 and UCP5. Together, these alterations are consistent with a progressive impairment of mitochondrial function during aging. Our findings further suggest that mitochondrial calcium handling, mitochondrial uncoupling pathways, and L-type calcium channel activity contribute to mitochondrial dysfunction in BAC-hR1441G DA neurons. These mechanisms may represent potential therapeutic targets for mitigating mitochondrial stress and preserving neuronal function in LRRK2-associated PD.

## Discussion

In the present study, we provide direct evidence that the pathogenic R1441G mutation is associated with progressive mitochondrial oxidative stress in identified nigrostriatal dopaminergic (DA) neurons and their terminals. Using two-photon ratiometric imaging of mitochondria-targeted roGFP, we define the temporal progression, cellular specificity, and molecular correlates of mitochondrial dysfunction in BAC-hR1441G mice. Our findings reveal an early increase in mitochondrial oxidative stress within striatal DA terminals that precedes substantial oxidative stress in SNc DA neuron somata, supporting a terminal-first pattern of pathology. Furthermore, we identify mitochondrial calcium overload and impaired UCP-dependent mitochondrial uncoupling as mechanisms associated with elevated oxidative stress and provide independent molecular support through spatial transcriptomic profiling.

A major advance of this study is the direct visualization of mitochondrial oxidative stress in identified nigrostriatal DA neurons across aging. Although mitochondrial dysfunction has long been implicated in PD, direct measurements of mitochondrial redox status in vulnerable DA neurons have been limited. Previous studies using two-photon imaging of mito-roGFP reporters in DJ-1-deficient mice demonstrated elevated mitochondrial oxidative stress in SNc DA neurons [5]. However, the temporal progression, regional specificity, and molecular correlates of mitochondrial oxidative stress in LRRK2-associated PD have remained poorly defined. We found that mitochondrial oxidation was elevated in striatal DA terminals as early as 3 months of age, whereas significant oxidative stress in SNc DA neuron somata emerged later and continued to increase with age. This terminal-first pattern is consistent with the “dying-back” model of Parkinson’s disease, in which synaptic and axonal dysfunction precede overt degeneration of neuronal cell bodies [31, 32]. Importantly, oxidative stress was not uniformly present across all DA neuron populations. VTA DA neurons did not exhibit increased mitochondrial oxidation, and neighboring TH-negative cells showed no increase in MitoSOX signal. Together, these findings indicate that mitochondrial oxidative stress preferentially affects vulnerable nigrostriatal DA neurons rather than reflecting a global oxidative state.

Our results further support a mutation-specific role of pathogenic LRRK2 signaling in mitochondrial dysfunction. BAC-hLRRK2-WT mice did not exhibit elevated oxidative stress, whereas pharmacological inhibition of LRRK2 kinase activity significantly reduced mitochondrial oxidation in BAC-hR1441G mice. These observations argue against a simple overexpression effect and support a pathogenic mechanism associated with the R1441G mutation. Although the R1441G mutation primarily affects the ROC GTPase domain, impaired GTP hydrolysis has been shown to enhance LRRK2 kinase-dependent signaling and alter multiple downstream pathways. Our findings therefore provide functional evidence linking pathogenic LRRK2 activity to mitochondrial oxidative stress in vulnerable DA neurons.

A second major finding is the identification of calcium dysregulation as a prominent feature of BAC-hR1441G DA neurons. Cytosolic and mitochondrial Ca^2 +^ levels were elevated, accompanied by increased MCU expression, while inhibition of L-type calcium channels reduced mitochondrial oxidation. Given the high energetic demands imposed by sustained calcium entry during autonomous pacemaking in SNc DA neurons, increased mitochondrial calcium uptake may promote oxidative stress. Although the present data do not establish a direct causal role for MCU, the concomitant increase in MCU expression and mitochondrial calcium accumulation suggests that altered mitochondrial calcium handling contributes to the oxidative phenotype observed in BAC-hR1441G mice.

Another important finding is the identification of impaired UCP-mediated mitochondrial uncoupling as a potential contributor to oxidative stress. BAC-hR1441G DA neurons exhibited markedly attenuated MMP flickering accompanied by reduced expression of UCP4 and UCP5. Because UCP-dependent uncoupling serves as an endogenous mechanism for limiting excessive ROS production, impaired uncoupling may further exacerbate oxidative stress and compromise mitochondrial homeostasis in vulnerable DA neurons.

Spatial transcriptomic profiling provided independent molecular support for these observations, revealing that dopaminergic neurons in BAC-hR1441G mice exhibit widespread upregulation of genes involved in OXPHOS, mitochondrial calcium transport, and ROS production. This analysis also highlighted the role of UCPs, which were downregulated in BAC-RG mice, leading to impaired MMP regulation and further exacerbation of oxidative stress. Our data suggest that UCP dysfunction contributes to ROS generation, offering a new mechanistic understanding of how LRRK2 mutations promote mitochondrial dysregulation. The convergence of imaging, physiological, biochemical, and transcriptomic findings strongly supports mitochondrial dysfunction as a central feature of the BAC-hR1441G phenotype. However, whether MCU and UCP4/UCP5 are directly regulated by pathogenic LRRK2 signaling remains unclear. These alterations may arise secondary to chronic calcium dysregulation and oxidative stress or reflect downstream transcriptional changes induced by mutant LRRK2. Future studies will be required to distinguish between these possibilities.

Given the strong link between LRRK2 mutations, oxidative stress, and calcium dysregulation, our findings highlight several potential therapeutic strategies. First, enhancing UCP function through pharmacological activation or gene therapy may help restore mitochondrial homeostasis and reduce ROS accumulation. Second, targeting MCU to limit mitochondrial calcium overload could mitigate oxidative stress-induced damage.

Importantly, our results support the use of isradipine (an L-type calcium channel blocker) as a potential neuroprotective strategy in LRRK2-related PD [5, 23]. While the STEADY-PD III clinical trial failed to demonstrate significant benefits in idiopathic PD, this is likely due to the intervention occurring at an irreversible stage of the disease, when over 80% of dopaminergic neurons have already been lost. The lack of efficacy does not rule out its potential in earlier stages. Future studies should focus on early intervention, particularly in high-risk individuals, such as genetically defined LRRK2-PD carriers, before significant neuronal loss occurs. Finally, future studies should assess whether selective MCU inhibitors or UCP activators/enhancers can provide neuroprotection in LRRK2-associated PD models.

Several limitations should be considered. Because BAC-hR1441G mice express mutant human LRRK2 above endogenous levels (approximately 2–3-fold) [33], some phenotypes may be influenced by transgene overexpression. However, BAC-hLRRK2-WT mice do not exhibit the dopaminergic release deficits and behavioral abnormalities [28]. Consistent with these reports, we found that BAC-LRRK2-WT mice did not exhibit elevated mitochondrial oxidative stress, arguing against a simple overexpression effect. Furthermore, LRRK2 kinase inhibition significantly reduced oxidative stress in BAC-hR1441G mice, supporting a mutation-specific mechanism. Future studies using knock-in models will be important to further validate these findings. Another limitation is that the molecular pathways linking the R1441G mutation to mitochondrial dysfunction remain incompletely defined. Although MCU expression was increased and UCP4/UCP5 expression was reduced in BAC-hR1441G mice, it remains unclear whether these changes are directly regulated by pathogenic LRRK2 signaling or arise secondary to chronic oxidative stress and calcium dysregulation. Future studies will be required to distinguish between these possibilities.

## Conclusion

Our study provides direct evidence that the R1441G mutation is associated with progressive mitochondrial oxidative stress in vulnerable nigrostriatal DA neurons. We demonstrate early oxidative stress in presynaptic terminals, followed by age-dependent mitochondrial dysfunction in SNc DA neurons, supporting a terminal-first pattern of pathology. In addition, disrupted calcium homeostasis, increased MCU expression, and impaired UCP-dependent mitochondrial uncoupling emerged as mechanisms associated with mitochondrial oxidative stress in BAC-hR1441G mice. These findings suggest that targeting MCU, L-type calcium channels, and UCPs may provide novel therapeutic strategies for LRRK2-associated PD.

## Electronic supplementary material

Below is the link to the electronic supplementary material.

Supplementary Material 1

Supplementary Material 2

Supplementary Material 3

Supplementary Material 4

Supplementary Material 5

Supplementary Material 6

Supplementary Material 7

## Data Availability

The datasets generated and/or analyzed during the current study are available from the corresponding author upon reasonable request.

## References

[1] Surmeier DJ. Determinants of dopaminergic neuron loss in Parkinson’s disease. FEBS J. 2018;285(19):3657–68. 10.1111/FEBS.14607. PMC6546423 30028088

[2] Lashuel HA, Petre BM, Wall J, Simon M, Nowak RJ, Walz T, et al. α-synuclein, especially the Parkinson’s disease-associated mutants, forms pore-like annular and tubular protofibrils. J Mol Biol. 2002;322(5):1089–102. 10.1016/S0022-2836(02)00735-0. 12367530

[3] Dauer W, Przedborski S. Parkinson’s disease. Neuron. 2003;39(6):889–909. 10.1016/S0896-6273(03)00568-3. 12971891

[4] Fearnley JM, Lees AJ. Ageing and parkinson’s disease: substantia nigra regional selectivity. Brain. 1991;114(5):2283–301. 10.1093/BRAIN/114.5.2283. 1933245

[5] Guzman JN, Sanchez-Padilla J, Wokosin D, Kondapalli J, Ilijic E, Schumacker PT, et al. Oxidant stress evoked by pacemaking in dopaminergic neurons is attenuated by DJ-1. Nature. 2010;468(7324):696–700. 10.1038/NATURE09536. PMC4465557 21068725

[6] Zimprich A, Biskup S, Leitner P, Lichtner P, Farrer M, Lincoln S, et al. Mutations in LRRK2 cause autosomal-dominant parkinsonism with pleomorphic pathology. Neuron. 2004;44(4):601–07. 10.1016/j.neuron.2004.11.005. 15541309

[7] West AB, Moore DJ, Biskup S, Bugayenko A, Smith WW, Ross CA, et al. Parkinson’s disease-associated mutations in leucine-rich repeat kinase 2 augment kinase activity. Proc Natl Acad Sci USA. 2005;102(46):16842–47. 10.1073/PNAS.0507360102. PMC1283829 16269541

[8] Kluss JH, Mamais A, Cookson MR. LRRK2 links genetic and sporadic Parkinson’s disease. Biochem Soc Trans. 2019;47(2):651–61. 10.1042/BST20180462. PMC6563926 30837320

[9] Healy DG, Falchi M, O’Sullivan SS, Bonifati V, Durr A, Bressman S, et al. Phenotype, genotype, and worldwide genetic penetrance of LRRK2-associated Parkinson’s disease: a case-control study. Lancet Neurol. 2008;7(7):583–90. 10.1016/S1474-4422(08)70117-0. PMC2832754 18539534

[10] Cookson MR. The role of leucine-rich repeat kinase 2 (LRRK2) in Parkinson’s disease. Nat Rev Neurosci. 2010;11(12):791–797. 10.1038/NRN2935. PMC4662256 21088684

[11] Cherra SJ, Steer E, Gusdon AM, Kiselyov K, Chu CT. Mutant LRRK2 elicits calcium imbalance and depletion of dendritic mitochondria in neurons. Am J Pathol. 2013;182(2):474–84. 10.1016/J.AJPATH.2012.10.027. PMC3562730 23231918

[12] Wang X, Yan MH, Fujioka H, Liu J, Wilson-Delfosse A, Chen SG, et al. LRRK2 regulates mitochondrial dynamics and function through direct interaction with DLP1. Hum Mol Genet. 2012;21(9):1931–44. 10.1093/HMG/DDS003. PMC3315202 22228096

[13] Singh A, Zhi L, Zhang H. LRRK2 and mitochondria: recent advances and current views. Brain Res. 2019;1702:96–104. 10.1016/J.BRAINRES.2018.06.010. PMC6281802 29894679

[14] Martin I, Kim JW, Lee BD, Kang HC, Xu JC, Jia H, et al. Ribosomal protein s15 phosphorylation mediates LRRK2 neurodegeneration in parkinson’s disease. Cell. 2014;157(2):472–85. 10.1016/j.cell.2014.01.064. PMC4040530 24725412

[15] Kim JW, Yin X, Martin I, Xiong Y, Eacker SM, Ingolia NT, et al. Dysregulated mRNA Translation in the G2019S LRRK2 and LRRK2 knock-out Mouse brains. eNeuro. 2021;8(6):8. 10.1523/ENEURO.0310-21.2021. PMC8638676 34759048

[16] Sanders LH, Laganière J, Cooper O, Mak SK, Vu BJ, Huang YA, et al. LRRK2 mutations cause mitochondrial DNA damage in iPSC-derived neural cells from Parkinson’s disease patients: reversal by gene correction. Neurobiol Disease. 2014;62:381–86. 10.1016/J.NBD.2013.10.013. PMC3877733 24148854

[17] Di Maio R, Hoffman EK, Rocha EM, Keeney MT, Sanders LH, De Miranda BR, et al. LRRK2 activation in idiopathic Parkinson’s disease. Sci Transl Med. 2018;10(451):1–13. 10.1126/scitranslmed.aar5429. PMC6344941 30045977

[18] Kim JW, Yin X, Jhaldiyal A, Khan MR, Martin I, Xie Z, et al. Defects in mRNA Translation in LRRK2-mutant hiPSC-Derived dopaminergic neurons lead to dysregulated calcium homeostasis. Cell STEM Cell. 2020;27(4):633–45.e7. 10.1016/J.STEM.2020.08.002. PMC7542555 32846140

[19] Sheu SS, Nauduri D, Anders MW. Targeting antioxidants to mitochondria: a new therapeutic direction. Biochim Et Biophys Acta (BBA) - Mol Basis Disease. 2006;1762(2):256–65. 10.1016/J.BBADIS.2005.10.007. 16352423

[20] Csordás G, Hajnóczky G. SR/ER–mitochondrial local communication: calcium and ROS. Biochim et Biophys Acta (BBA) - Bioenerg. 2009;1787(11):1352–62. 10.1016/J.BBABIO.2009.06.004. PMC2738971 19527680

[21] Chan CS, Gertler TS, Surmeier DJ. Calcium homeostasis, selective vulnerability and Parkinson’s disease. Trends Neurosciences. 2009;32(5):249–56. 10.1016/J.TINS.2009.01.006. PMC4831702 19307031

[22] Surmeier DJ, Guzman JN, Sanchez-Padilla J, Schumacker PT. The role of calcium and mitochondrial oxidant stress in the loss of substantia nigra pars compacta dopaminergic neurons in Parkinson’s disease. Neuroscience. 2011;198:221–31. 10.1016/j.neuroscience.2011.08.045. PMC3244353 21884755

[23] Guzman JN, Ilijic E, Yang B, Sanchez-Padilla J, Wokosin D, Galtieri D, et al. Systemic isradipine treatment diminishes calcium-dependent mitochondrial oxidant stress. J Clin Invest. 2018;128(6):2266–80. 10.1172/JCI95898. PMC5983329 29708514

[24] Li Y, Liu W, Oo TF, Wang L, Tang Y, Jackson-Lewis V, et al. Mutant LRRK2R1441G BAC transgenic mice recapitulate cardinal features of Parkinson’s disease. Nat Neurosci. 2009;12(7):826-828. 10.1038/nn.2349. PMC2845930 19503083

[25] Kim-Han JS, Dugan LL. Mitochondrial uncoupling proteins in the central nervous System. Antioxid Redox Signal. 2005;7(9–10):1173–81. 10.1089/ARS.2005.7.1173. https://home.liebertpub.com/ars. 16115020

[26] Zhi L, Qin Q, Muqeem T, Seifert EL, Liu W, Zheng S, et al. Loss of PINK1 causes age-dependent decrease of dopamine release and mitochondrial dysfunction. Neurobiol Aging. 2019;75:1–10. 10.1016/J.NEUROBIOLAGING.2018.10.025. PMC6778692 30504091

[27] Santini E, Turner KL, Ramaraj AB, Murphy MP, Klann E, Kaphzan H. Mitochondrial superoxide contributes to hippocampal synaptic dysfunction and memory deficits in angelman syndrome model mice. J Neurosci. 2015;35(49):16213–20. 10.1523/JNEUROSCI.2246-15.2015. PMC4682786 26658871

[28] Qin Q, Zhi LT, Li XT, Yue ZY, Li GZ, Zhang H. Effects of LRRK2 inhibitors on nigrostriatal dopaminergic neurotransmission. CNS Neurosci Ther. 2017;23(2):162–73. 10.1111/cns.12660. PMC5248597 27943591

[29] Moore DJ, West AB, Dawson VL, Dawson TM. Molecular pathophysiology of parkinson’s disease. Annu Rev Neurosci. 2005;28:57–87. 10.1146/annurev.neuro.28.061604.135718. 16022590

[30] Tagliaferro P, Burke RE. Retrograde axonal degeneration in parkinson disease. J Parkinson’s Disease. 2016;6(1):1-15. 10.3233/JPD-150769. PMC4927911 27003783

[31] Burke RE, O’Malley K. Axon degeneration in Parkinson’s disease. Exp Neurol. 2013;246:72–83. 10.1016/J.EXPNEUROL.2012.01.011. PMC3340476 22285449

[32] Moon HE, Paek SH. Mitochondrial dysfunction in Parkinson’s disease. Exp Neurobiol. 2015;24(2):103–116. 10.5607/EN.2015.24.2.103. PMC4479806 26113789

[33] Tagliaferro P, Kareva T, Oo TF, Yarygina O, Kholodilov N, Burke RE. An early axonopathy in a hLRRK2(R1441G) transgenic model of Parkinson's disease. Neurobiol Disease. 2015;82:359–71. 10.1016/j.nbd.2015.07.009. PMC4640977 26192625

