# Supplementary material for: The human LRRK2-R1441G mutation drives age-dependent oxidative stress and mitochondrial dysfunction in dopaminergic neurons
Source: Mol Neurodegener. 2026 Jul 7;21:69. doi: 10.1186/s13024-026-00969-7 (PMC13689286; doi:10.1186/s13024-026-00969-7)
Supplement: Supplementary file 1 [file 13024_2026_969_MOESM1_ESM.pdf]

Extended Data Figures:

Extended Data Fig.1: **Mitochondrial respiration remains intact in BAC-hR1441G mice at 3 and 6 months of age**

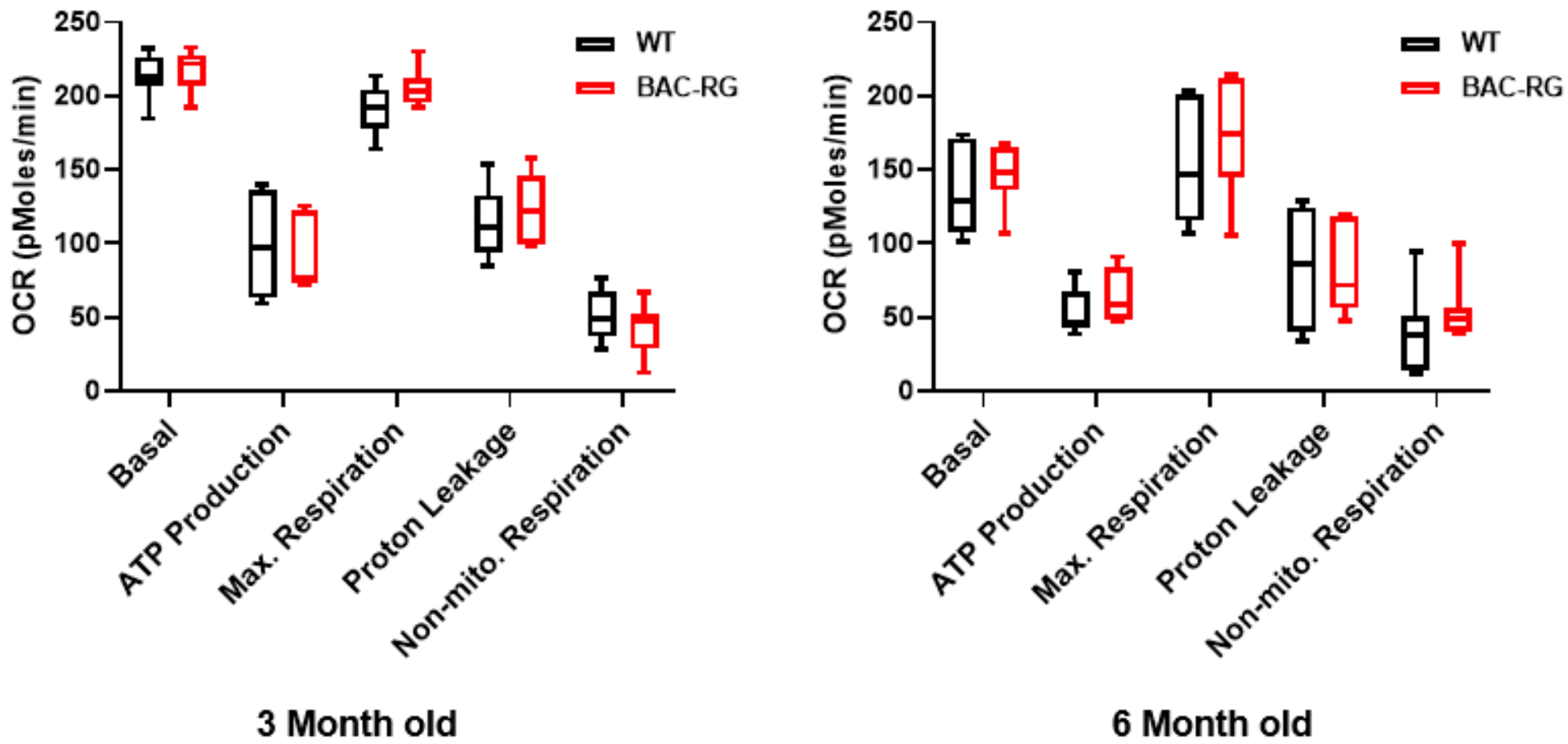

Extended Data Fig.2: **Excitation spectra of mitochondrial roGFP in SNc DA neurons under basal, reduced, and oxidized conditions**

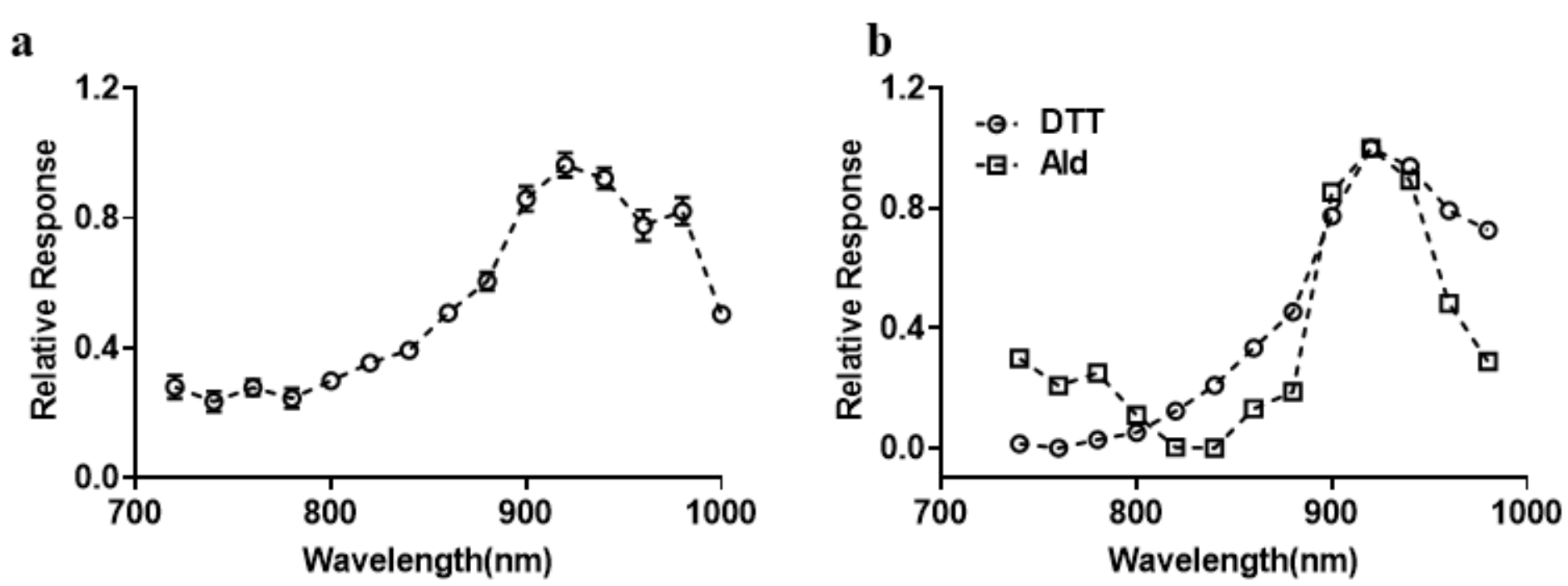

(**a**) Excitation spectrum of mitochondria-targeted roGFP expressed in SNc DA neurons under physiological (basal) conditions. (**b**) Excitation spectra following treatment with the reducing agent dithiothreitol (DTT, 2 mM) and the oxidizing agent aldrithiol (Ald, 100 µM), respectively.

Extended Data Fig.3: Dynamic response of mitochondrial roGFP to sequential reduction (DTT) and oxidation (ALD).

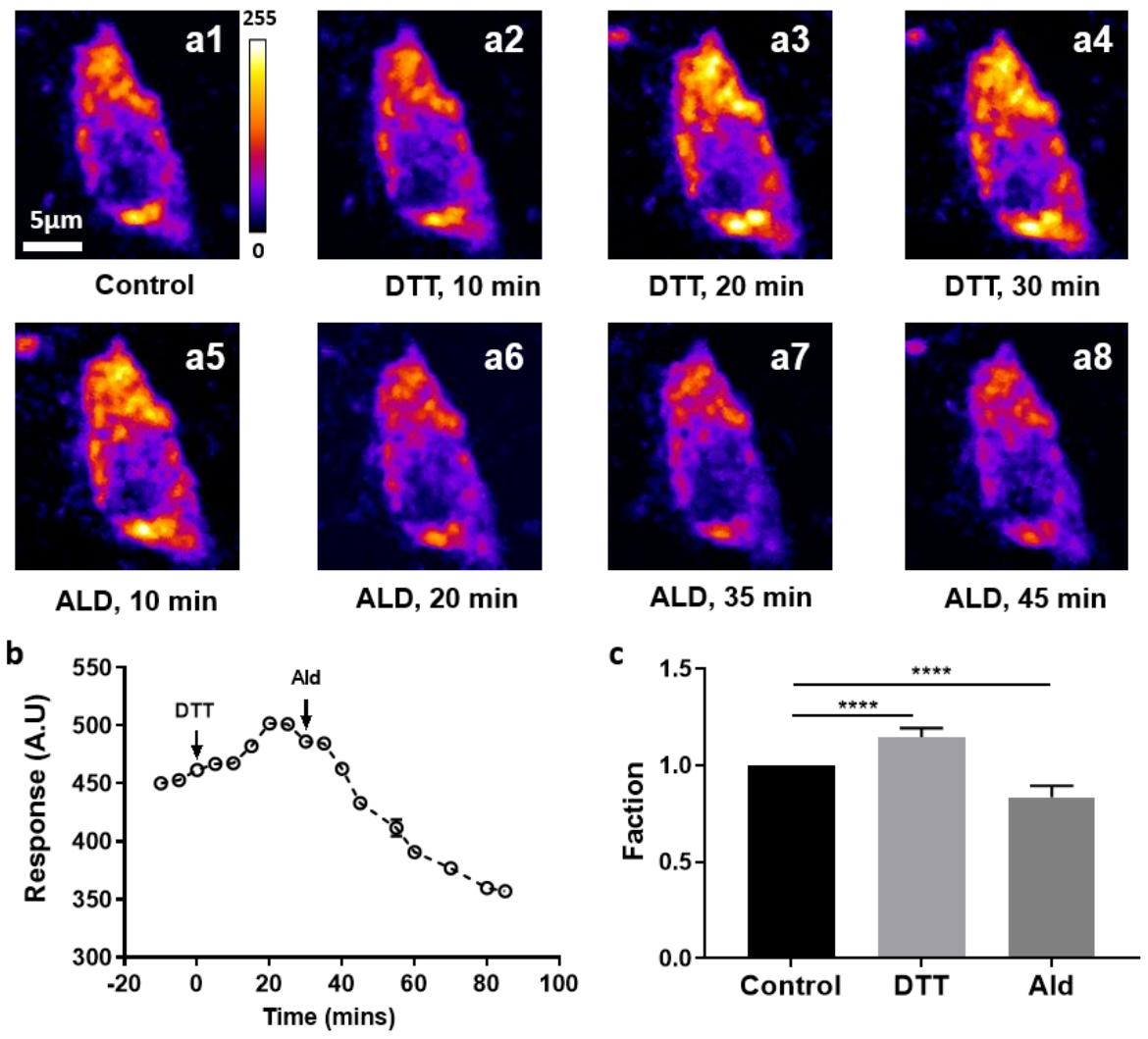

(**a1-a8**) Representative images of mitochondrial roGFP fluorescence in SNc DA neurons at the indicated time points during sequential treatment with DTT (2 mM) and Ald (100 µM). (**b**) Quantification of roEGFP fluorescence response to DTT and Ald treatments. (**c**) Summary data showing roGFP responses under basal, reduced, and oxidized conditions following sequential DTT and Ald treatments (N = 4, n = 6).

Extended Data Fig.4: Validation of ratiometric roGFP imaging.

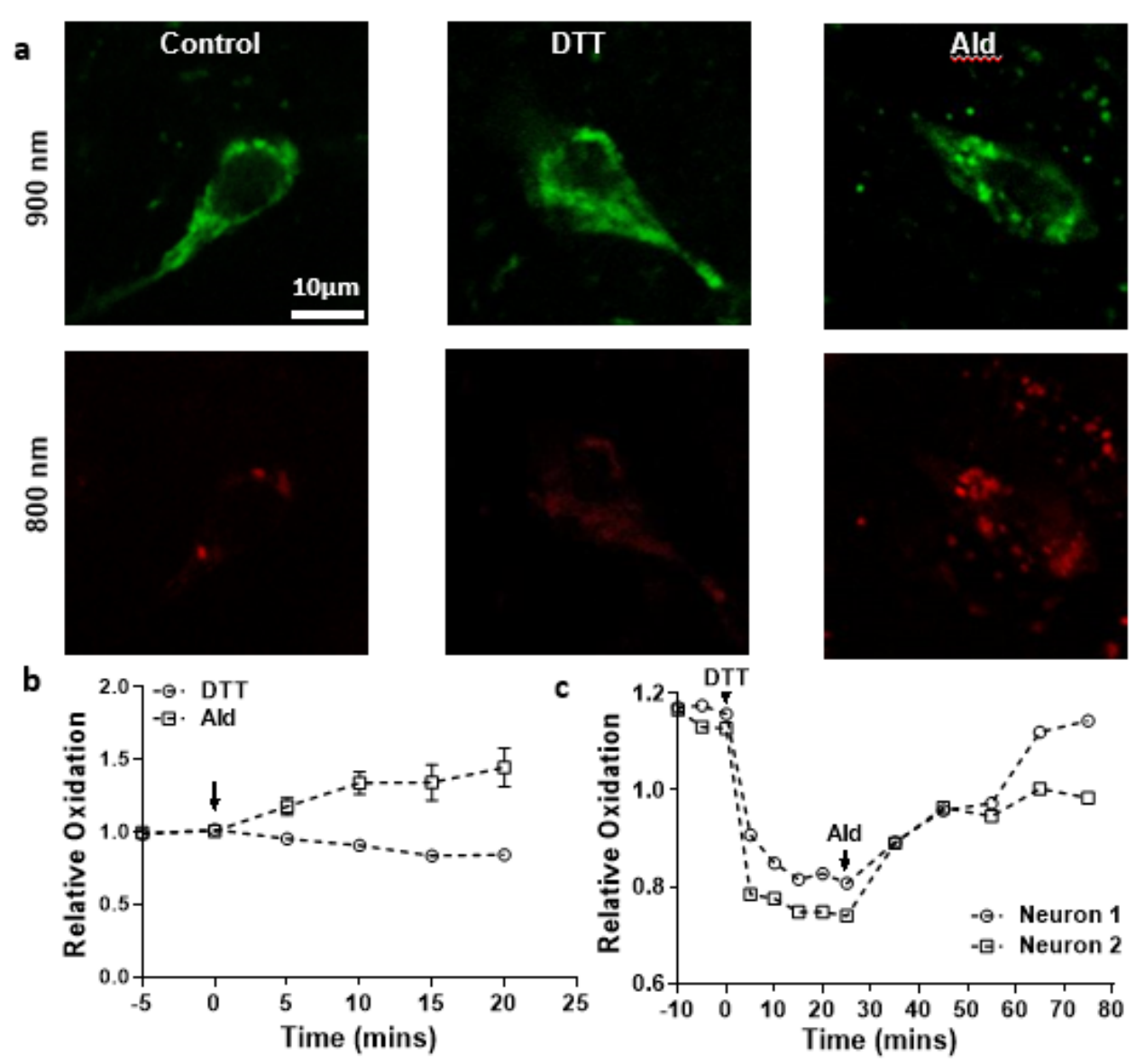

(**a**) Representative roEGFP images under different conditions (left: physiological; middle: fully reduced with DTT; right: fully oxidized with Ald) captured at two excitation wavelengths (900 nm and 800 nm). (**b**) Validation of the ratiometric method for measuring roEGFP response to DTT and Ald treatments respectively. (c) Validation of the ratiometric imaging measuring roGFP responses during sequential DTT and Ald treatments.

Extended Data Fig.5: Oxidative levels in SNc DA neurons of WT and BAC-hR1441G mice at three different ages

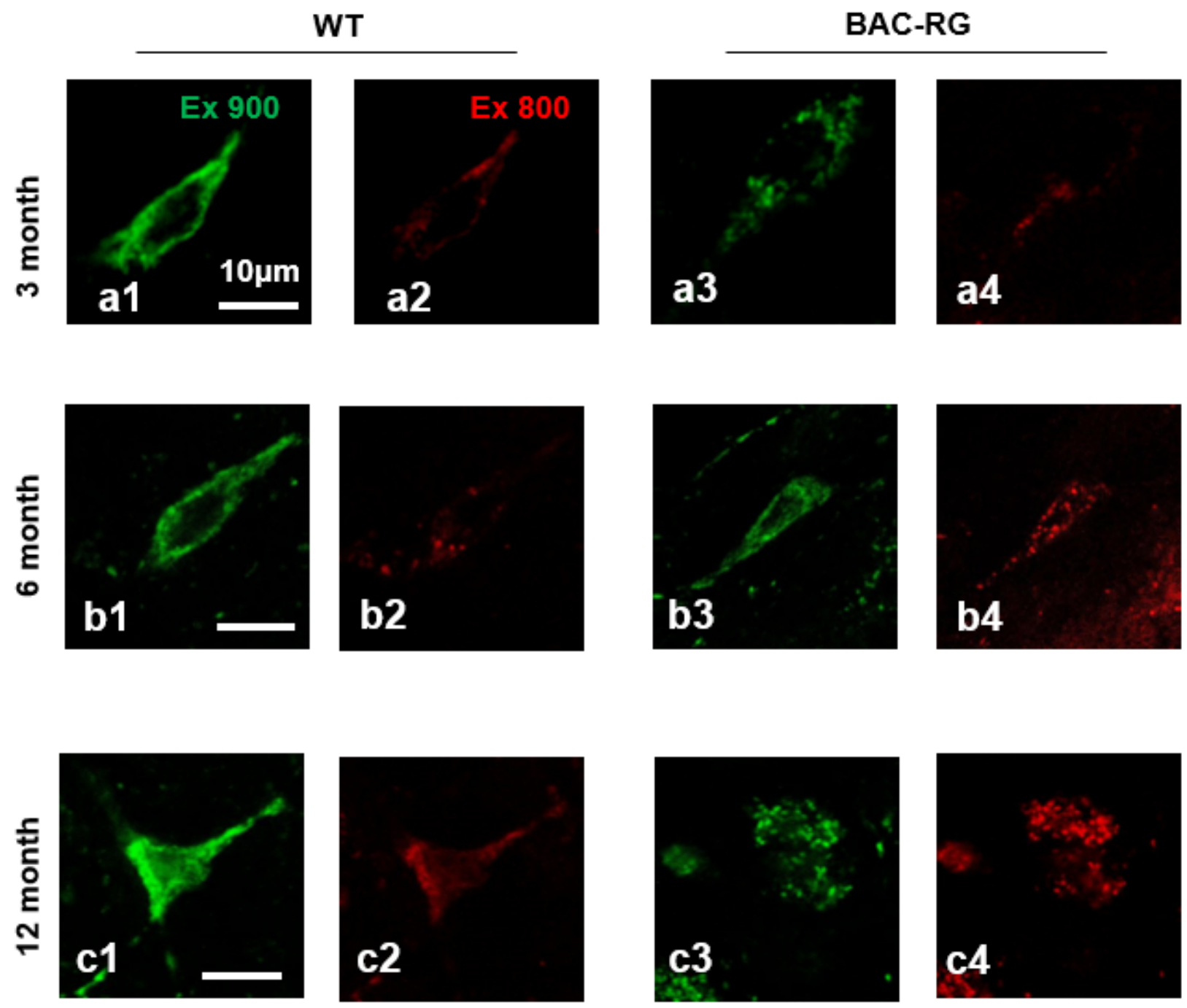

(**a1–a4, b1–b4, c1–c4**) Representative images of mitochondrial roGFP expressed in SNc DA neurons from WT and BAC-hR1441G mice, acquired at two excitation wavelengths (900 nm and 800 nm) across three age groups: 3, 6, and 12 months, respectively.

Extended Data Fig.6: **Oxidative levels in dSTR DA terminals of WT and BAC-hR1441G mice at three different ages**

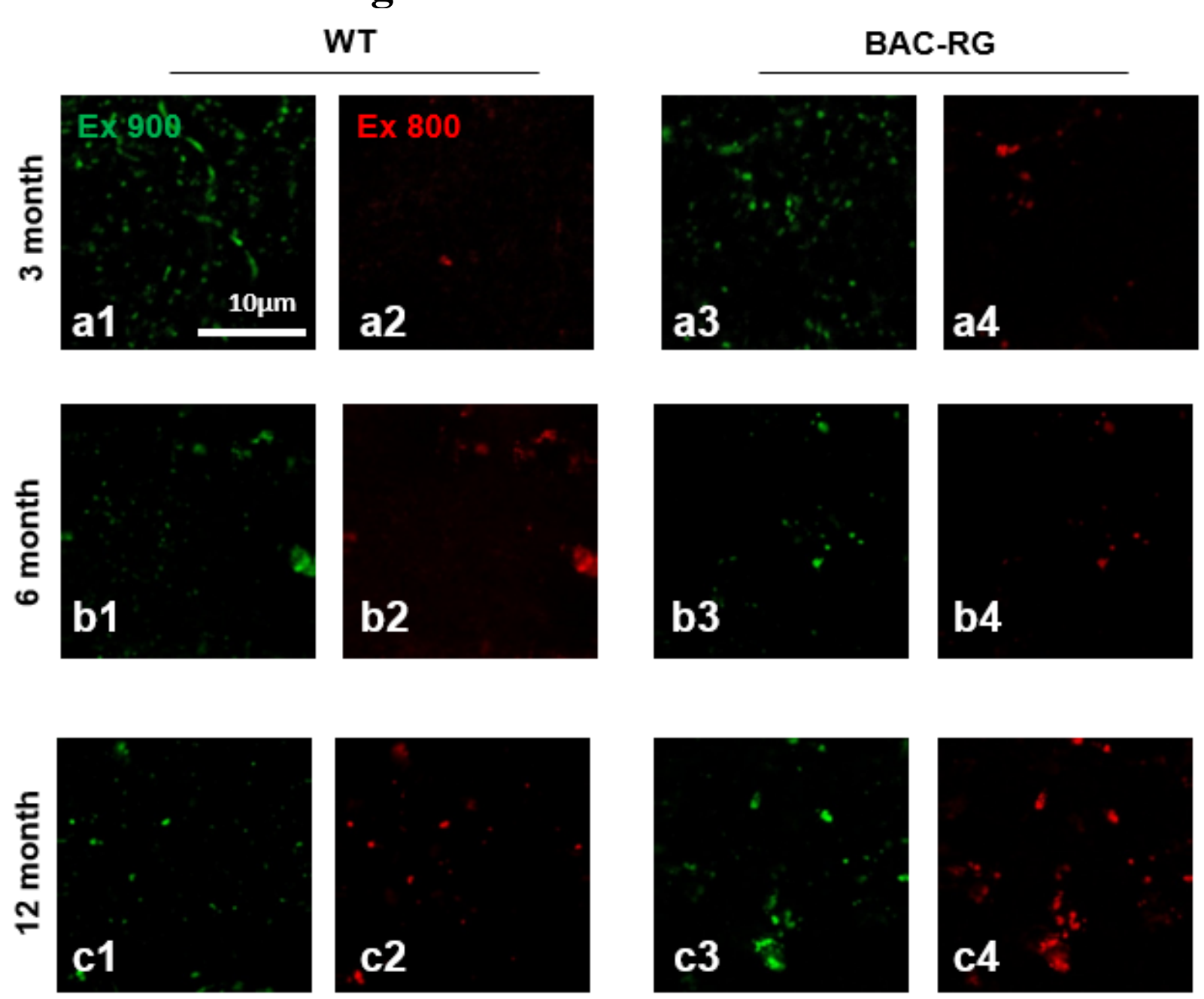

(**a1–a4, b1–b4, c1–c4**) Representative images of mitochondrial roGFP expression in dSTR DA terminals from WT and BAC-hR1441G mice, acquired at two excitation wavelengths (900 nm and 800 nm) across three age groups: 3, 6, and 12 months.

Extended Data Fig.7: **Overexpression of WT human LRRK2 does not elevate mitochondrial oxidative levels**

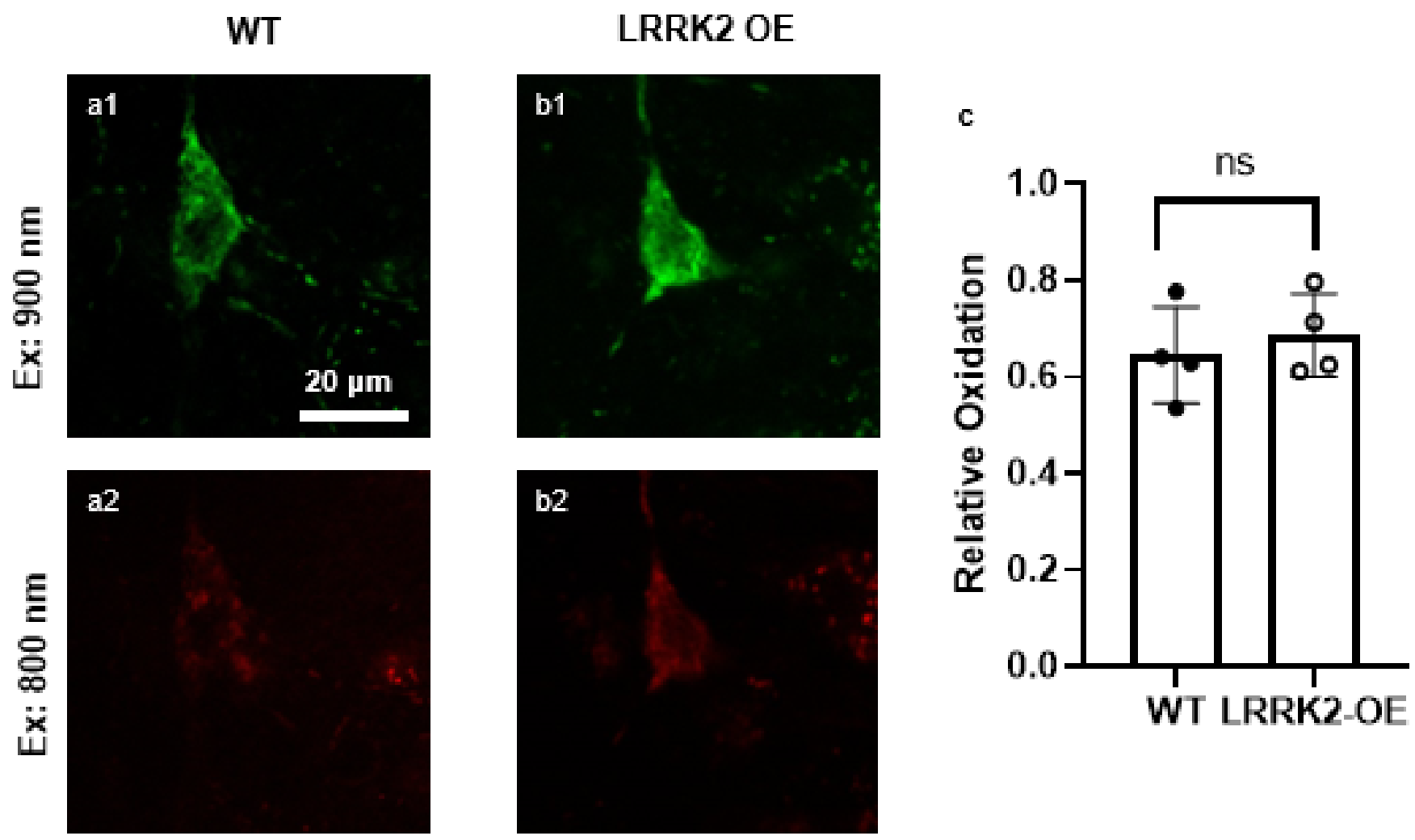

(**a1–a2, b1–b2**) Representative images of mitochondrial roGFP fluorescence in SNc DA neurons from WT and BAC-hLRRK2-WT mice. Images were acquired at excitation wavelengths of 800 nm and 900 nm, respectively. (c) Quantification of mitochondrial oxidative stress using the fluorescence ratio (F800/F900). No significant difference was detected between WT and BAC-hLRRK2-WT mice, indicating that overexpression of WT human LRRK2 does not increase mitochondrial oxidative stress in SNc DA neurons (4 pairs; Mann–Whitney test, P = 0.8857).

Extended Data Fig.8: **GNE-7915 reduces mitochondrial oxidative stress in BAC-hR1441G brain slices.**

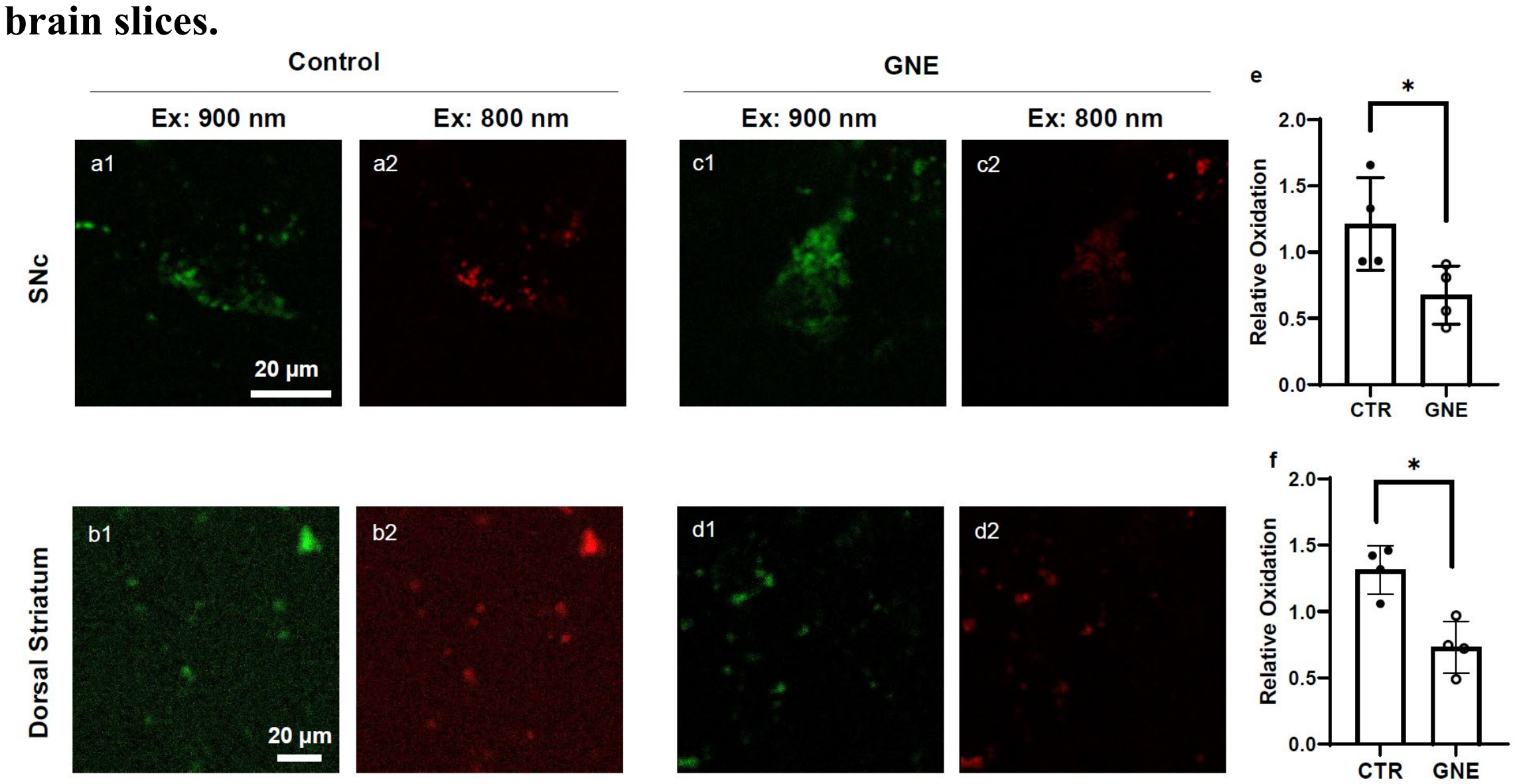

(a1-a2, **b1-b2**, **c1-c2**, **d1-d2)** Representative two-photon images of mitochondrial roGFP fluorescence in SNc DA neurons and dSTR DA terminals from BAC-hR1441G mice following treatment with GNE-7915 (3 μM). Mitochondrial redox status was assessed by ratiometric imaging using excitation wavelengths of 800 nm and 900 nm. (**e, f**) Quantification of mitochondrial oxidative stress using the fluorescence ratio (F800/F900). GNE-7915 treatment significantly reduced mitochondrial oxidative stress in both SNc DA neurons and dSTR DA

terminals of BAC-hR1441G mice (4 pairs, Control (CTR) vs GNE, Mann–Whitney test, DA neurons in the SNc, * P = 0.0286; DA terminals in the dSTR, * P = 0.0286)

Extended Data Fig.9: **Mitochondrial oxidative stress is not elevated in VTA DA neurons of 3- and 6-month-old BAC-hR1441G mice**

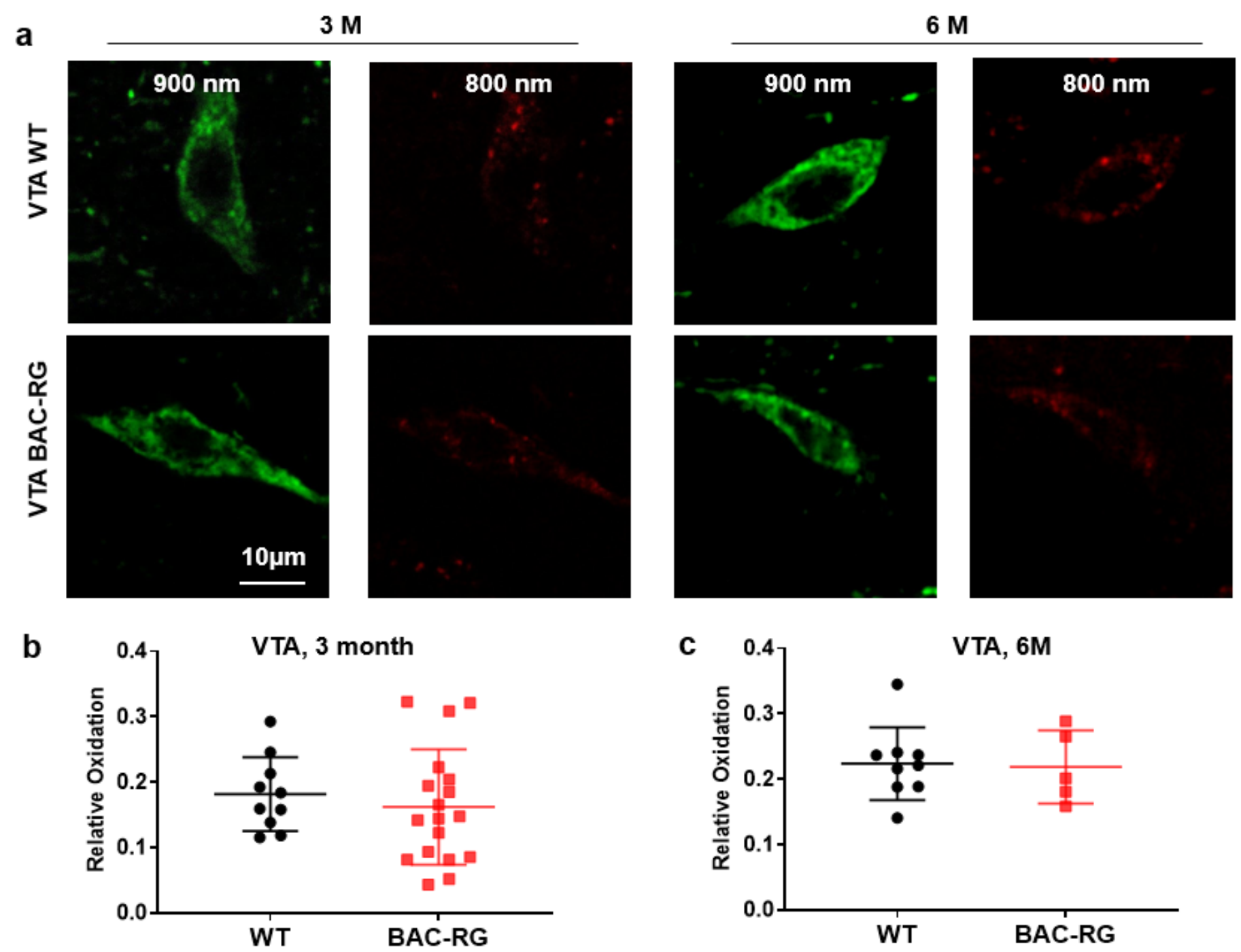

(**a**) Representative ratiometric roGFP images of VTA DA neurons from WT and BAC-hR1441G mice at 3 and 6 months of age. Images were acquired at excitation wavelengths of 900 nm (green) and 800 nm (red). (**b**) Quantification of mitochondrial oxidation in VTA DA neurons from 3-month-old mice (3 pairs; WT: n = 10 neurons; BAC-hR1441G: n = 18 neurons, Mann–Whitney test, P > 0.05). (**c**) Quantification of mitochondrial oxidation in VTA DA neurons from 6-month-old mice (3 pairs; WT: n = 10 neurons; BAC-hR1441G: n = 6 neurons, Mann–Whitney test, P > 0.05).

Extended Data Fig.10: **MitoSOX Red imaging of mitochondrial superoxide levels in the dorsal striatum**

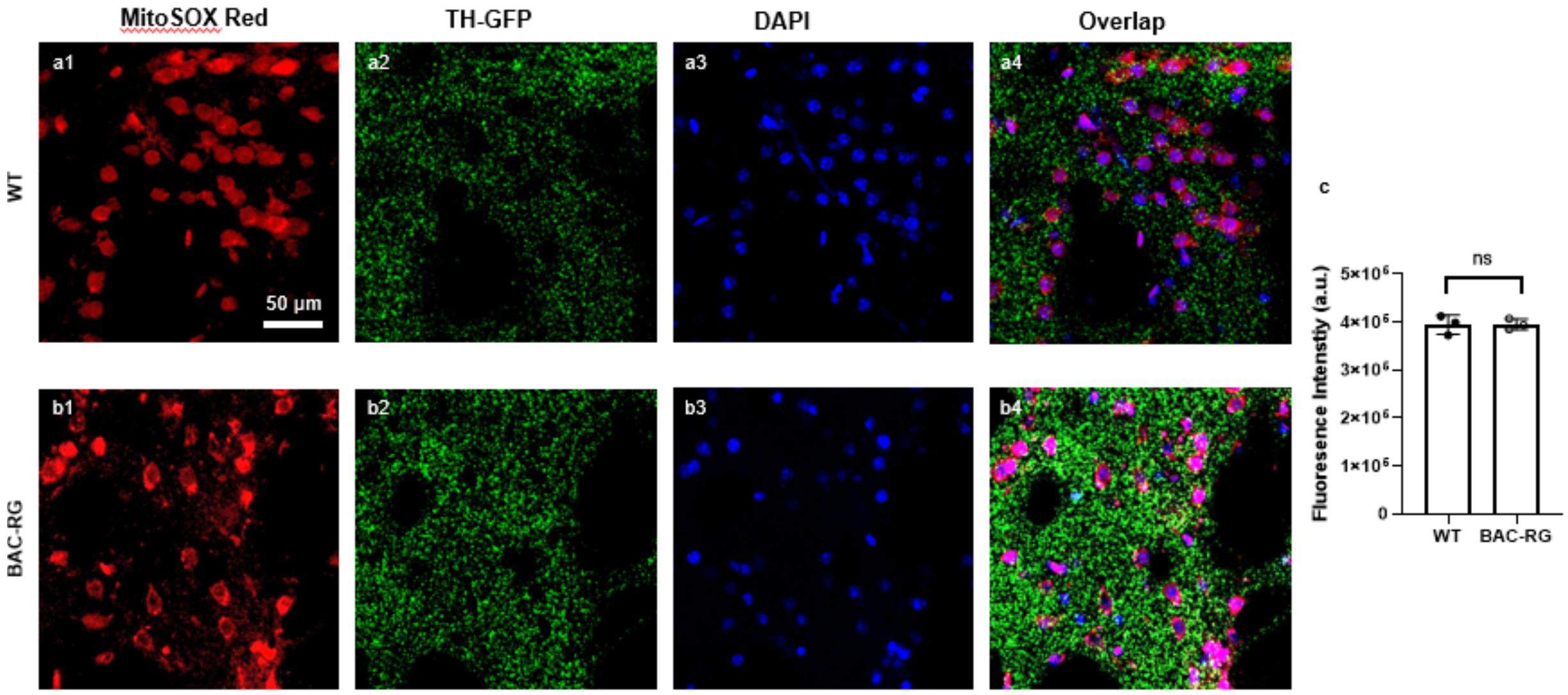

(**a1–a4, b1–b4**) Representative images of the dorsal striatum from 7-month-old WT and BAC-hR1441G mice labeled with MitoSOX Red (red), TH-GFP (green), and DAPI (blue). Merged images are shown. (**c**) Quantification of MitoSOX fluorescence intensity in the dorsal striatum. No significant difference in mitochondrial superoxide levels was detected between WT and BAC-hR1441G mice (3 pairs; Mann–Whitney test, $P > 0.9999$).

Extended Data Fig.11: **MitoSOX Red imaging of mitochondrial superoxide levels in the SN**

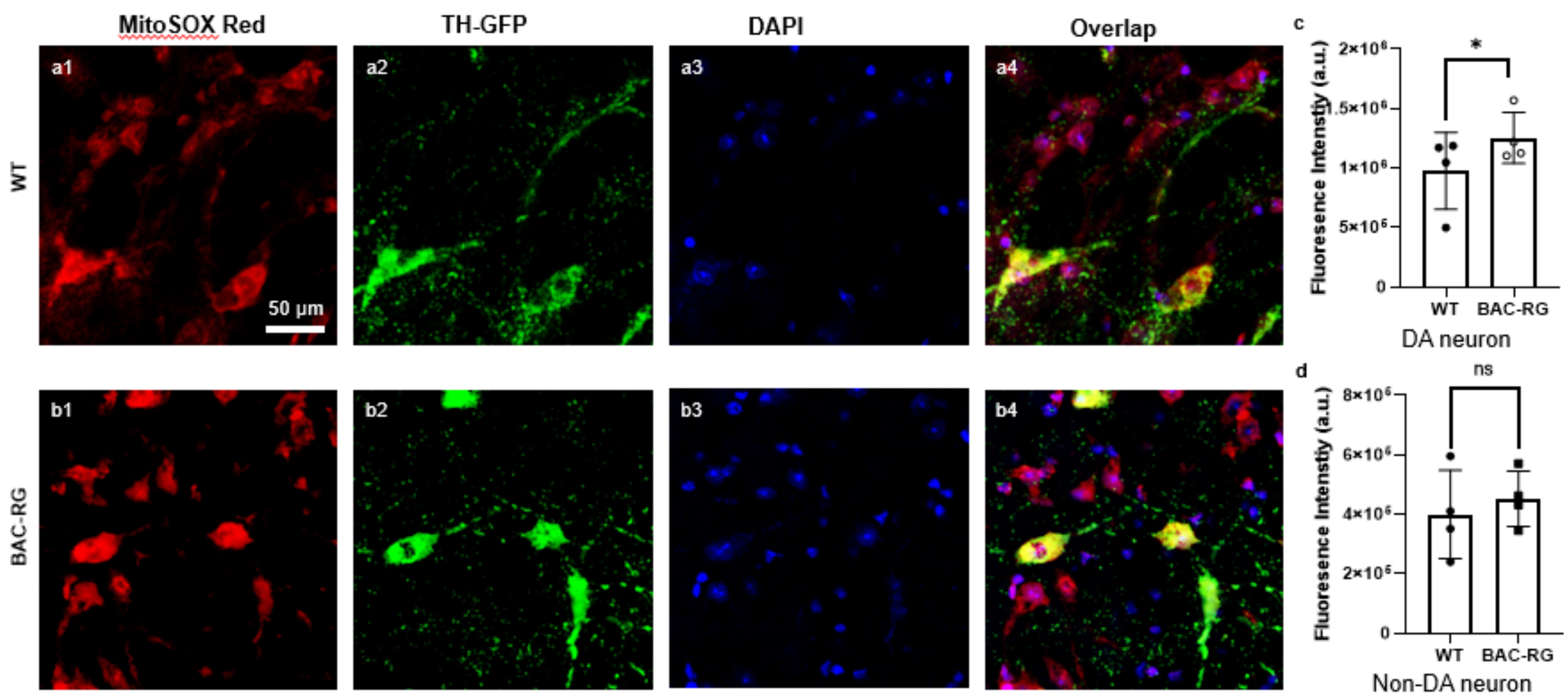

(**a1-a4, b1-b4**) Representative images of the SNc from 7-month-old WT and BAC-hR1441G mice labeled with MitoSOX Red (red), TH-GFP (green), and DAPI (blue). Merged images are shown. (**c, d**) Quantification of MitoSOX fluorescence intensity revealed significantly increased mitochondrial superoxide levels in DA neurons, but not in non-DA cells, within the SNc of BAC-hR1441G mice compared with WT controls (4 pairs; Mann–Whitney test: DA neurons, *$P = 0.0205$; non-DA cells, $P = 0.6875$).

Extended Data Fig.12: **Identification of TMRM-labeled SNc DA neurons based on TH-GFP expression**

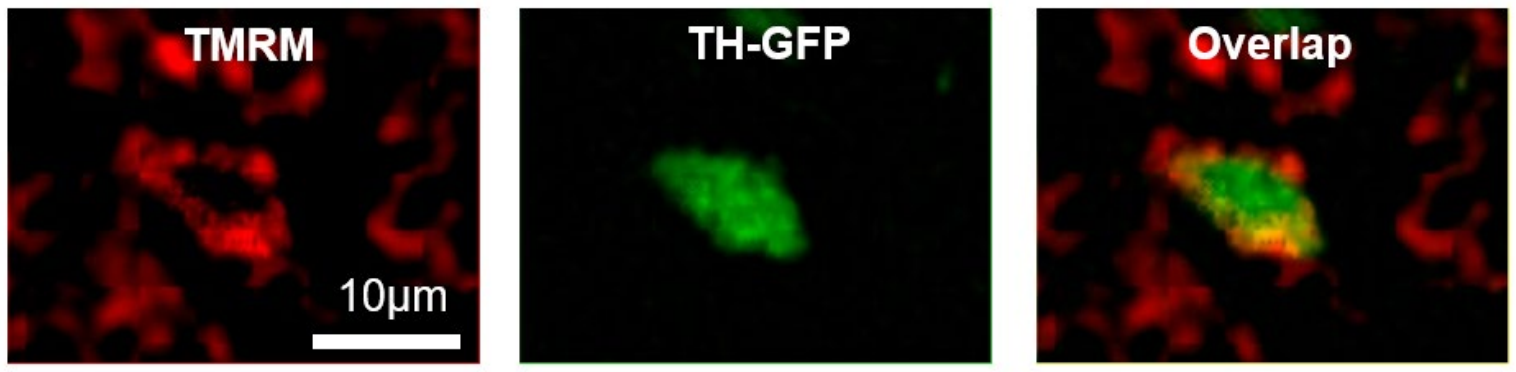

Extended Data Fig.13: **The R1441G mutation accelerates the age-dependent decline in MMP flickering in SNc DA neurons**

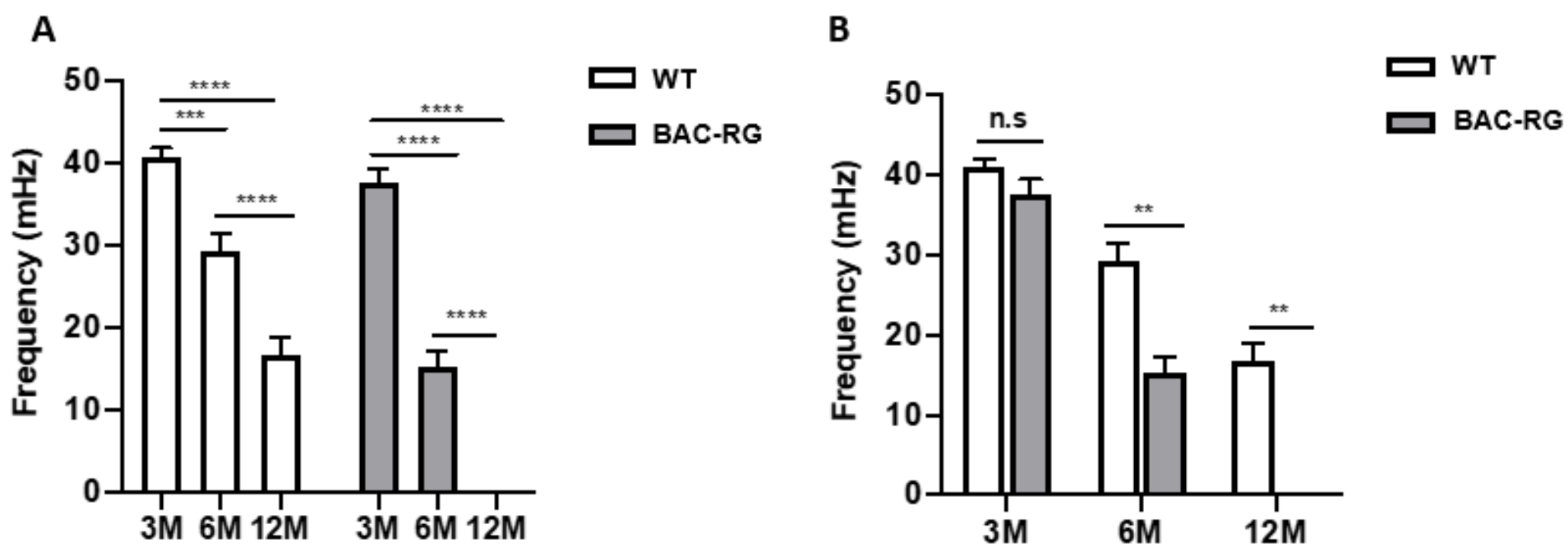

(**a**) MMP flickering frequency decreases with age in SNc DA neurons. (**b**) Quantification of MMP flickering frequency in WT and BAC-hR1441G mice at 3, 6, and 12 months of age (3 m: 3 pairs, WT n = 6, BAC-hR1441G n = 6; 6 m: 4 pairs, WT n = 8, BAC-hR1441G n = 8; 12 m: 3 pairs, WT n = 7, BAC-hR1441G n = 6).

Extended Data Fig.14: **Isradipine and genipin reduce MMP flickering in SNc DA neurons**

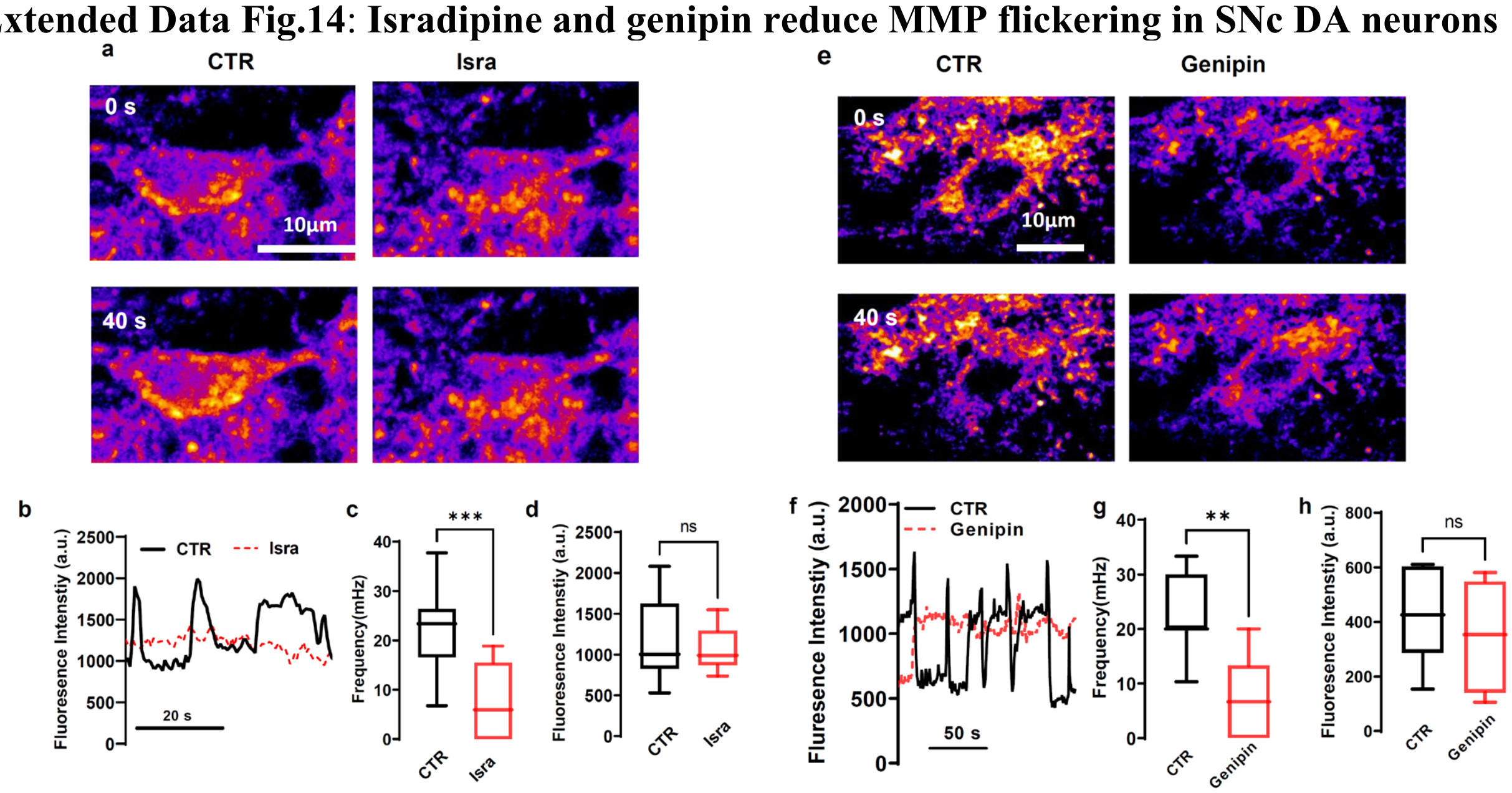

(**a**) Representative TMRM fluorescence images of SNc DA neurons before and after isradipine treatment (10 μM, 30 min). (**b**) Representative traces showing that isradipine significantly reduces MMP flickering frequency. (**c, d**) Quantification of the effects of isradipine on MMP

flickering frequency and amplitude, demonstrating a significant reduction in flickering events following treatment (N = 4 mice, n = 8 neurons; Mann–Whitney test, ***P = 0.0010). (**e**) Representative TMRM fluorescence images of SNc DA neurons before and after genipin treatment (10 μM, 30 min). (**f**) Representative traces showing that genipin significantly reduces MMP flickering frequency. (**g, h**) Quantification of the effects of genipin on MMP flickering frequency and amplitude, demonstrating a significant reduction in flickering events following treatment (N = 6 mice, n = 10 neurons; Mann–Whitney test, **P = 0.0052).

Extended Data Fig.15: **KEGG pathway enrichment analysis of differentially expressed genes (DEGs) between BAC-hR1441G and WT DA neurons**

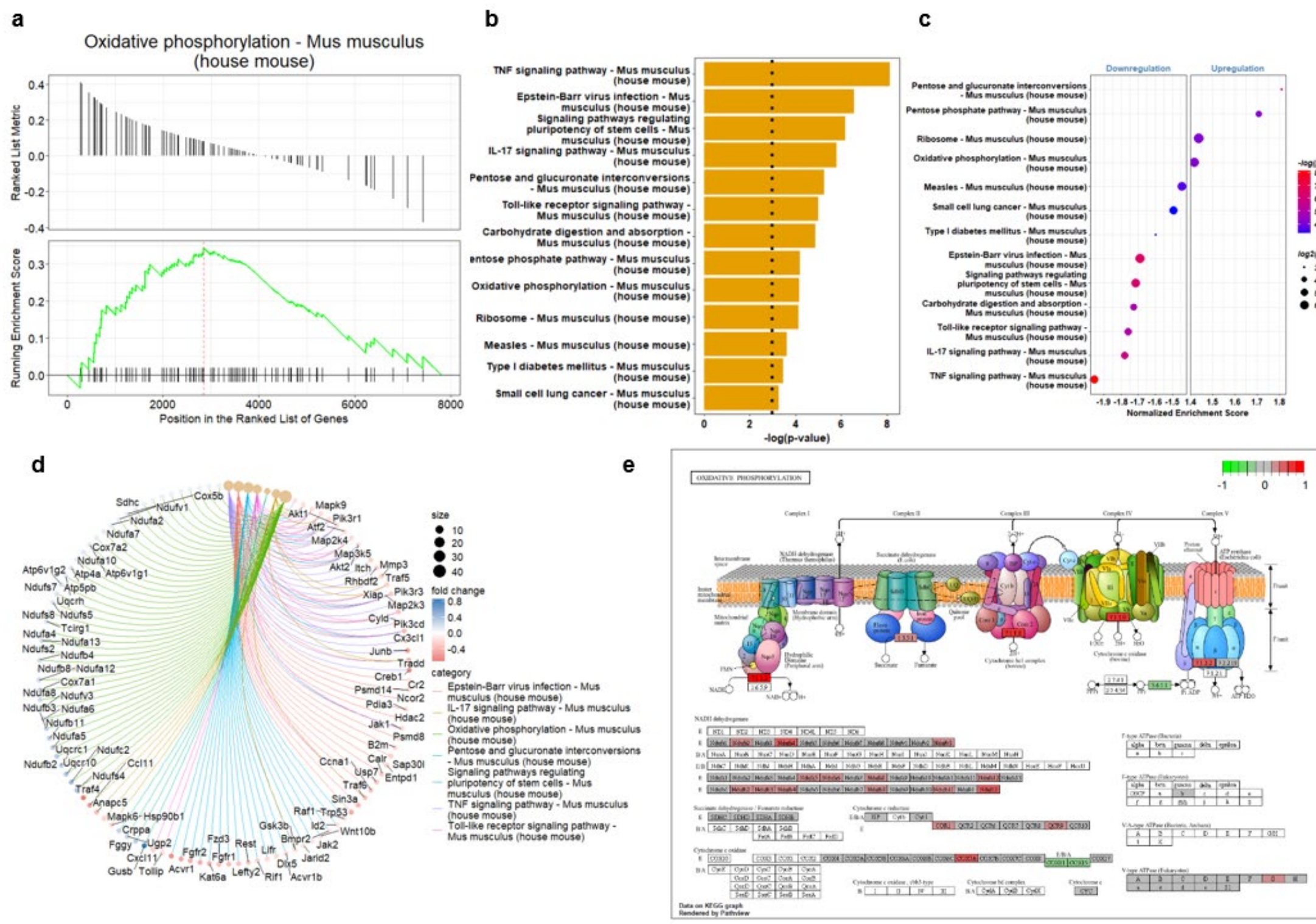

(**a**) Enrichment plot comparing BAC-hR1441G to WT in the KEGG gene set. The peak in the plot reflects the upregulation of gene sets associated with the enrichment term: Oxidative phosphorylation – Mus musculus (house mouse). (**b**) Pathway analysis of differentially expressed genes (DEGs) between BAC-hR1441G and WT. (**c**) Gene set enrichment analysis (GSEA) of the KEGG gene set based on DEGs from BAC-hR1441G versus WT, represented as a hierarchically clustered heatmap. The color scale corresponds to the -Log_{10} of the p-value for the enrichment score, and the size of the circles corresponds to the number of leading genes in each enriched term. (**d**) CNET plot from gene set enrichment analysis, illustrating the complex associations between genes and significantly enriched terms. Node size is proportional to the significance of the enrichment (adjusted p-value), while the color of the gene nodes represents fold change values. Edges indicate the associations between genes and the enriched terms. (**e**) Visualization of the Oxidative phosphorylation enrichment term, highlighting upregulated and downregulated genes.

Extended Data Video Legends

Extended Data Video 1: Representative movies of mitochondrial membrane potential in SNc DA neurons from WT (A) and BAC-RG (B) mice.

Extended Data Video 2: MPG treatment reduced the MMP flickering frequency of SNc DA neurons.

Extended Data Video 3: L-type calcium channel blocker, isradipine, reduced the MMP flickering frequency of SNc DA neurons.

Extended Data Video 4: The uncoupling protein (UCP) inhibitor genipin reduced the MMP flickering frequency in SNc DA neurons.
